# P2X7R deficiency alleviates cardiac senescence by enhancing mitophagy via the HuR/TRIM26/NR4A1 axis

**DOI:** 10.1002/ctm2.70621

**Published:** 2026-02-26

**Authors:** Yixin Zhou, Xin Zhong, Zhijie Mao, Yunxuan Chen, Jincheng Xing, Jiaxu Shen, Wenli Zhang, Ji Zhang, Jiaxuan Mei, Zhentong Yang, Zhuoqun Wang, Bozhi Ye, Jiahui Lin, Yonghua Wang, Zhouqing Huang

**Affiliations:** ^1^ Department of Cardiology The First Affiliated Hospital of Wenzhou Medical University, The Key Laboratory of Cardiovascular Disease of Wenzhou Wenzhou China; ^2^ Department of Cardiology The Second Affiliated Hospital of Jiaxing University Jiaxing China; ^3^ Department of Cardiovascular Medicine Ruijin Hospital Shanghai Jiao Tong University School of Medicine Shanghai China; ^4^ Department of Nephrology The First Affiliated Hospital of Wenzhou Medical University Wenzhou China; ^5^ Department of Geriatric Medicine The First Affiliated Hospital of Wenzhou Medical University Wenzhou China; ^6^ Department of Physical Education Wenzhou Medical University Wenzhou China

**Keywords:** ageing, cardiac remodelling, human antigen R, mitophagy, NR4A1, P2X7R, TRIM26

## Abstract

**Background:**

Ageing is a significant risk factor for pathophysiological alterations in the heart, but the intrinsic mechanisms by which these occur have yet to be fully elucidated. Purinergic 2×7 receptor (P2X7R) is important for the pathogenesis of numerous cardiovascular diseases; nevertheless, its function in the process of cardiac ageing remains uncertain.

**Methods:**

This study utilised P2X7R knockout (P2X7R^−^/^−^) mice. An ageing model was established by either maintaining mice until they reached 20 months of age or performing chronic subcutaneous injection of D‐galactose (D‐gal). Recombinant adeno‐associated virus serotype 9 (AAV9) was employed to achieve cardiac‐specific overexpression of P2X7R and nuclear receptor subfamily 4 group A member 1 (NR4A1). Cardiac function and histopathological changes in cardiac tissues were evaluated. Transcriptome sequencing was further applied to elucidate the potential mechanisms of P2X7R in cardiac senescence.

**Result:**

Our result show that serum levels of P2X7R increase with advancing age in humans and that P2X7R expression is upregulated during cardiac senescence in mice. P2X7R deficiency alleviates ageing‐related cardiac dysfunction, senescence phenotypes and impaired mitophagy. Cardiomyocyte‐specific overexpression of P2X7R with AAV9 exacerbates the myocardial dysfunction, senescence phenotype and mitophagy disruption induced by D‐gal. Mechanistically, P2X7R promotes human antigen R (HuR) nucleocytoplasmic shuttling in ageing hearts, thereby increasing the mRNA stability of tripartite motif containing 26 (TRIM26) and the expression of the E3 ubiquitin ligase TRIM26. TRIM26 subsequently mediates NR4A1 ubiquitination, leading to its proteasomal degradation, which subsequently suppresses mitophagy in cardiomyocytes and ultimately accelerates cardiac ageing.

**Conclusions:**

Our findings provide valuable insights into the role of P2X7R in cardiac ageing and identify the HuR/TRIM26/NR4A1 axis as a key signalling pathway through which P2X7R regulates cardiac ageing.

## Graphical abstract

P2X7R regulates the nucleoplasmic translocation of HuR, thereby enhancing the stability of the E3 ubiquitin ligase TRIM26. TRIM26 directly binds to NR4A1 and promotes its ubiquitination, thereby suppressing mitophagy and increasing reactive oxygen species (ROS) production. Consequently, this triggers inflammasome activation and enhances the secretion of senescence‐associated secretory phenotype (SASP) factors, ultimately contributing to cardiac dysfunction in cardiac senescence.

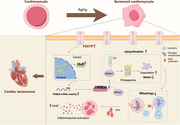

## 1 INTRODUCTION

Ageing, a universal biological process that occurs in all higher organisms, leads to the progressive degeneration of major organ systems. With the ongoing demographic shift towards an ageing population,^1^ the prevalence of cardiac dysfunction among older adults continues to increase. Extensive research has demonstrated that senescence occurs in all major cardiac cell types and contributes to the gradual deterioration of cardiac structure and function, which is associated with atrial fibrillation, cardiac hypertrophy, fibrosis^2^, ^3^ and increased susceptibility to heart failure with reduced ejection fraction.^4^, ^5^, ^6^ Therefore, a thorough understanding of the complex molecular mechanisms underlying cardiac ageing is essential for identifying novel therapeutic targets to preserve or improve cardiac function in elderly individuals.

A hallmark of cardiac ageing is mitochondrial dysfunction, particularly impaired mitophagy, a selective autophagic process responsible for the degradation of damaged mitochondria.^7^ With advancing age, the efficiency of oxidative phosphorylation decreases, leading to disrupted adenosine triphosphate (ATP) production and increased reactive oxygen species (ROS) generation.^8^ Increased ROS levels induce oxidative damage to mitochondrial DNA, proteins and lipids, thereby disrupting mitochondrial dynamics and further hindering mitophagy. Accumulating evidence indicates that defective mitophagy in cardiomyocytes plays a critical role in the pathogenesis of age‐related heart failure.^9^ Both mitophagy and overall autophagic flux are frequently suppressed in the ageing myocardium,^10^ whereas appropriately increased mitophagy helps delay the progression of heart failure.^9^ Thus, timely and efficient removal of dysfunctional and aged mitochondria is essential for preserving cardiomyocyte function and viability.

The purinergic receptor family, particularly the P2X purine receptors, comprises ATP‐dependent trimeric ion channels that are widely expressed in nearly all human tissues. These receptors regulate calcium signalling and inflammatory responses^11^, ^12^ and play critical roles in the pathogenesis of numerous diseases. Notably, purinergic 2×7 receptor (P2X7R) is characterised by its unique ability to form large‐pore membrane channels, activate the NLRP3 inflammasome, promote the release of proinflammatory mediators and trigger cell death.^13^ Our research team focuses on the role and regulatory mechanisms of P2X7R in the pathogenesis of cardiovascular diseases, including pathological cardiac hypertrophy,^14^ diabetic cardiomyopathy^15^ and atrial fibrillation,^16^ and has identified P2X7R as a significant contributor to these conditions. Therefore, P2X7R holds promise as a key molecular target for the identification of therapeutic interventions for cardiovascular diseases. However, research on its role and underlying mechanisms in cardiac ageing remains limited.

In the present study, we investigated P2X7R alterations in human serum and ageing mice and demonstrated that P2X7R deletion exerts cardioprotective effects against cardiomyocyte ageing by enhancing cardiac mitophagy. Transcriptome sequencing (RNA‐seq) of heart tissue revealed nuclear receptor subfamily 4 group A member 1 (NR4A1) as a potential downstream effector of P2X7R, which is consistent with its established role in regulating ageing and mitophagy.^17^, ^18^ By integrating RNA‐seq and RT‒qPCR data, we further revealed that the E3 ubiquitin ligase tripartite motif containing 26 (TRIM26) directly binds to NR4A1 and promotes its degradation. Moreover, we found that P2X7R stabilises TRIM26 mRNA and upregulates TRIM26 expression in aged cardiomyocytes by triggering nucleocytoplasmic shuttling of the RNA‐binding protein human antigen R (HuR). Our study identifies a novel HuR/TRIM26/NR4A1 signalling pathway through which P2X7R regulates mitophagy in ageing cardiomyocytes, providing a valuable foundation for future research aimed at developing targeted therapies for cardiac senescence.

## 2 MATERIALS AND METHODS

### 2.1 Experimental animals

All animal procedures were carried out in compliance with the ethical standards and guidelines set forth by the US National Institutes of Health. The study protocol was approved by the Laboratory Animal Ethics Committee of the First Affiliated Hospital of Wenzhou Medical University (approval document no. WYYY‐IACUC‐AEC‐2021‐0262). The mice were maintained under controlled conditions with a 12‐h light/dark cycle and at an ambient temperature of 22°C–23°C and a relative humidity ranging from 40% to 60%.

To explore the function of P2X7R in cardiac ageing, male C57BL/6 wild‐type (WT) mice and global germline P2X7R knockout (P2X7R‐KO) male mice (GemPharmatech Co. Ltd.) were utilised. The animals were randomly allocated into one of three groups (*n* = 6 per group): (1) WT‐young, (2) WT‐old, and (3) P2X7R^−/−^‐old. The mice received sterile water and standard chow (MediScience Diets Co. Ltd.) until they reached the target ages: 3 months for the WT‐young group and 24 months for the WT‐old and P2X7R^−/−^‐old groups. Following anaesthesia with isoflurane, cardiac ultrasound was performed. Euthanasia was subsequently conducted via an overdose of carbon dioxide. Serum and cardiac tissue samples were collected for further analysis.

To achieve cardiomyocyte‐specific overexpression of P2X7R and NR4A1, male mice were administered adeno‐associated virus serotype 9 (AAV9) carrying a cardiac‐specific promoter (cTnT). The constructs included AAV9‐cTnT encoding an empty vector (EV), P2X7R (AAV9‐cTnT‐P2X7R^oe^) or NR4A1 (AAV9‐cTnT‐NR4A1^oe^) (GV571; Genechem Co. Ltd.). WT C57BL/6 mice were divided into the following groups (*n* = 6 per group): (1) WT + AAV9‐cTnT‐EV + D‐gal, (2) WT + AAV9‐cTnT‐P2X7R^oe^ + D‐gal, (3) WT + AAV9‐cTnT‐NR4A1^oe^ + D‐gal, and (4) WT + AAV9‐cTnT‐P2X7R^oe^ + AAV9‐cTnT‐NR4A1^oe^ + D‐gal. AAV9 was delivered via tail vein injection (2 × 10^11^ vg/mouse/month, two injections). Two weeks later, the mice received daily subcutaneous injections of D‐galactose (D‐gal, 300 mg/kg/day; Sangon Biotech) for 6 weeks to establish a D‐gal‐induced ageing model. Control animals were administered an equivalent volume of saline.

Cardiac remodelling models induced by angiotensin II (Ang II) and a high‐fat diet/streptozotocin (HFD/STZ) were established on the basis of previously described methods. For the Ang II‐induced hypertrophy model, 12 WT C57BL/6J mice were randomly assigned to two groups (*n* = 6 per group). Ang II was continuously infused subcutaneously at 1 µg/kg/min using osmotic minipumps (Alzet Model 1004), while control animals received saline; both treatments were performed for 4 weeks. In the HFD/STZ model, 8‐week‐old C57BL/6 mice were fed either a HFD (45% fat, 35% carbohydrate, 20% protein) or standard chow for 24 weeks. After 8 weeks of HFD feeding, insulin deficiency was induced by intraperitoneal administration of low‐dose STZ (40 mg/kg) for 3 consecutive days. The control mice received citrate buffer on the same schedule.

### 2.2 Enzyme‐linked immunosorbent assay

To measure the serum level of P2X7R in human individuals of varying ages, a Purinergic Receptor P2X, Ligand Gated Ion Channel 7 (P2RX7) enzyme‐linked immunosorbent assay kit (Affandie, A101865) was used. All procedures were conducted in accordance with the instructions provided with the reagent kit.

Human serum samples were obtained from patients of different ages (young, middle‐aged and elderly). The clinical characteristics of the patients are presented in Table S3. Notably, all protocols involving human serum samples were meticulously reviewed and formally approved by the Ethics Committee of the First Affiliated Hospital of Wenzhou Medical University (approval document no. KY2024‐R246).

### 2.3 Statistical analysis

The data are presented as the mean ± standard error of the mean. Numerical values indicate the number of biological replicates. Owing to the central limit theorem, in vitro data (each being an average of many cells) were assumed to follow a normal distribution. For in vivo data, the Shapiro‒Wilk test was applied to evaluate normality, and a *p*‐value >.05 was interpreted as an approximately normal distribution. Student's *t* test was applied for comparisons between two groups and one‐way ANOVA followed by Tukey's post hoc test was employed for comparisons among multiple groups. Statistical significance was set at *p* < .05. All the statistical analyses were performed using SPSS version 23.0 software and GraphPad Prism 10.0 software.

An extended Materials and Methods section is available in the Supporting Information.

## 3 RESULTS

### 3.1 P2X7R expression in the serum of elderly humans and in heart tissues of aged mice

**FIGURE 1:**
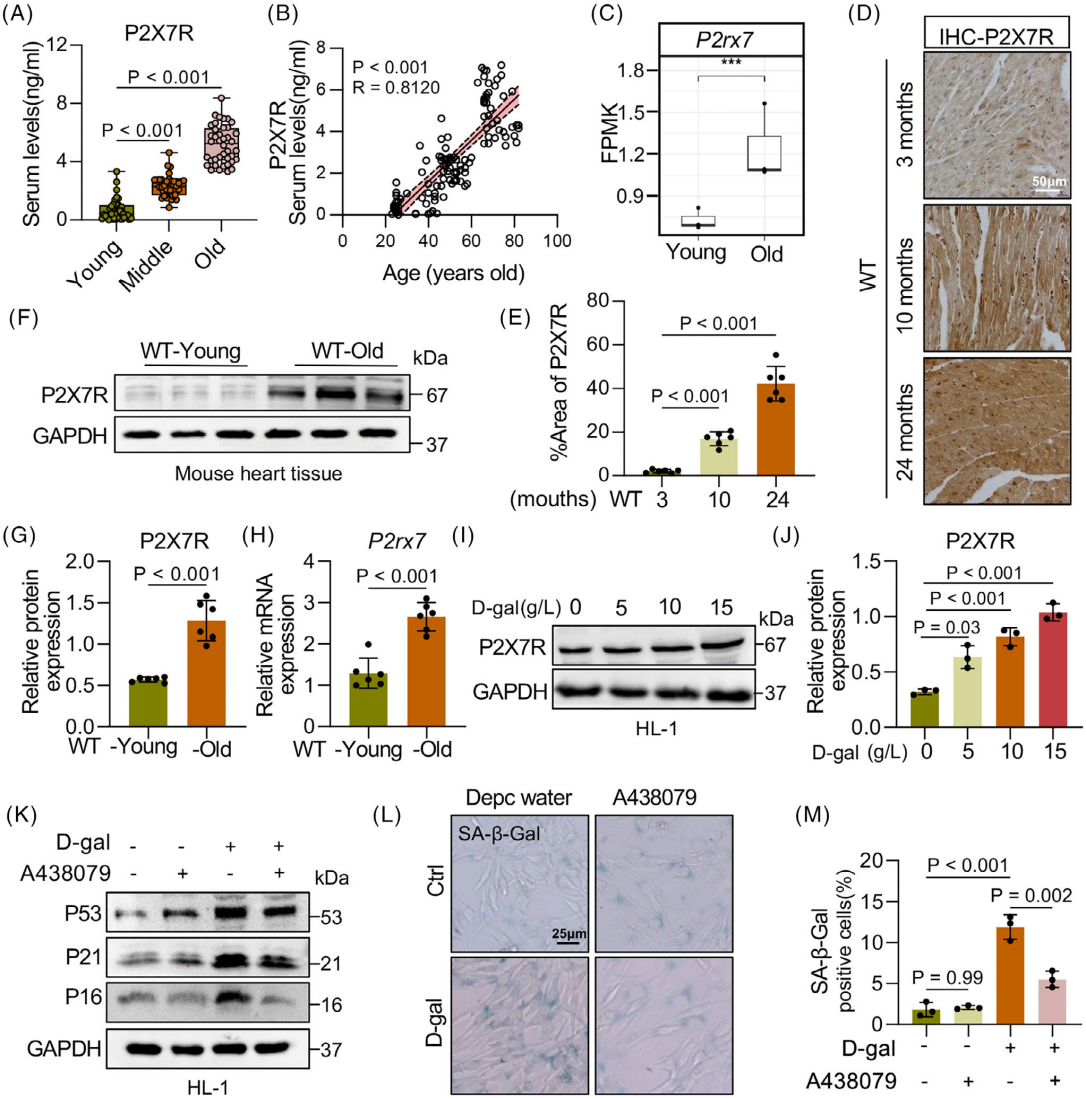
Purinergic 2×7 receptor (P2X7R) expression in the serum of elderly humans and heart tissues of aged mice. (A) P2X7R levels in the serum of humans in three age groups: young, middle‐aged and elderly (*n* = 40). (B) Correlation between P2X7R serum expression and human age. (C) Transcriptome sequencing data showing differential P2X7R gene expression in the heart tissues of young and old mice. (D) Representative images of P2X7R immunoreactivity in the myocardia of 3‐, 10‐ and 24‐month‐old mice (scale bar, 50 µm) (*n* = 6). (E) Semiquantitative analysis of the P2X7R area ratio in (D) (*n* = 6). (F) P2X7R expression in the myocardial tissue of the wild type (WT)‐young and WT‐old groups. GAPDH was used as a loading control (*n* = 6). (G) Densitometric quantification of the immunoblots in (F) (*n* = 6). (H) mRNA levels of P2X7R in the myocardial tissue of the WT‐young and WT‐old groups (*n* = 6). (I) Representative Western blot analysis of P2X7R levels in HL‐1 cells treated with 0, 5, 10 or 15 g/L D‐galactose (D‐gal). GAPDH was used as a loading control (*n* = 3). (J) Densitometric quantification of the immunoblots in (I) (*n* = 3). (K) Representative Western blot analysis of tumour protein 53 (P53), CDN1A (P21) and p16INK4a (P16) levels in D‐gal‐induced HL‐1 cells pretreated with A438079 or DMSO. GAPDH was used as a loading control (*n* = 3). (L) Representative senescence‐associated β‐galactosidase (SA‐β‐Gal) staining images of HL‐1 cells (*n* = 3). (M) Percentages of SA‐β‐Gal+ cells in (L) were quantified (*n* = 3). Adjusted *p*‐values are provided in the case of multiple group comparisons. P2X7R, purinergic receptor P2X, ligand‐gated ion channel 7; young, 3 months old; old, 24 months old; A438079, P2X7R inhibitor. DMSO, Dimethyl Sulfoxide; GAPDH, Glyceraldehyde‐3‐Phosphate Dehydrogenase.

To study the effect of P2X7R on ageing, we first assessed P2X7R levels in serum samples from 120 individuals of different ages (under 44, 45–59 and 60–90 years, which are equivalent to the young, middle and elderly life stages in this study, respectively) and observed that P2X7R expression was significantly upregulated in an age‐dependent manner (Figure 1A and Table S3). Furthermore, after adjusting for potential confounders, including inflammatory markers, specific cardiovascular conditions and medication history, subgroup analysis and multivariate ANOVA revealed that age remained an independent factor positively correlated with serum P2X7R levels (Figure 1B and Table S4). To determine the primary organ involved, we utilised naturally aged mice and assessed the expression of P2X7R across multiple tissues and found that P2X7R expression was especially elevated in the heart (Figure S1A). Given that circulating P2X7R levels may be affected by various physiological and pathological factors, we sought to investigate the direct link between serum P2X7R levels and cardiac P2X7R expression. To this end, we measured P2X7R levels in both the cardiac tissue and serum of naturally ageing mice. P2X7R expression was significantly elevated in both the heart and serum of aged mice compared with young controls (Figure S1B,C). Importantly, a significant positive correlation was observed between cardiac and serum P2X7R levels (Figure S1D). Next, we analysed high‐throughput transcriptome sequencing data from the hearts of 3‐ and 24‐month‐old mice and detected significantly greater expression of P2X7R in aged mice than in young mice (Figure 1C). Consistently, increased levels of P2X7R were observed at both the protein and mRNA levels in the cardiac tissues of aged mice (Figure 1D‒H). These results revealed that P2X7R levels were significantly elevated in the hearts of aged mice, suggesting that P2X7R may be involved in the induction of cardiac ageing.

To investigate the expression and cellular origin of P2X7R, we treated primary rat cardiomyocytes, cardiac fibroblasts and macrophages with D‐gal to induce cellular senescence and observed that P2X7R expression was significantly upregulated in cardiomyocytes, whereas no marked increase in P2X7R expression was detected in fibroblasts or macrophages (Figure S1E). Moreover, double‐immunofluorescence staining of cardiac tissues from aged mice revealed that P2X7R colocalised with α‐actinin + cardiomyocytes (Figure S1F). These data indicated that P2X7R is expressed mainly in cardiomyocytes during cardiac senescence. We subsequently treated HL‐1 cardiomyocytes with different concentrations of P2X7R and found that P2X7R expression increased in a concentration‐dependent manner (Figures 1I,J and S1G). In addition, we preliminarily investigated whether A438079, a pharmacological inhibitor of P2X7R, markedly suppressed the expression of p21, p53 and p16, which are markers of cellular senescence (Figures 1K and S1H). This effect was confirmed by staining for senescence‐associated β‐galactosidase (SA‐β‐Gal) (Figure 1L,M). These findings suggest that P2X7R may serve as a key regulator of cellular senescence.

### 3.2 P2X7R deficiency delays ageing in mice and alleviates ageing‐induced cardiac remodelling

**FIGURE 2:**
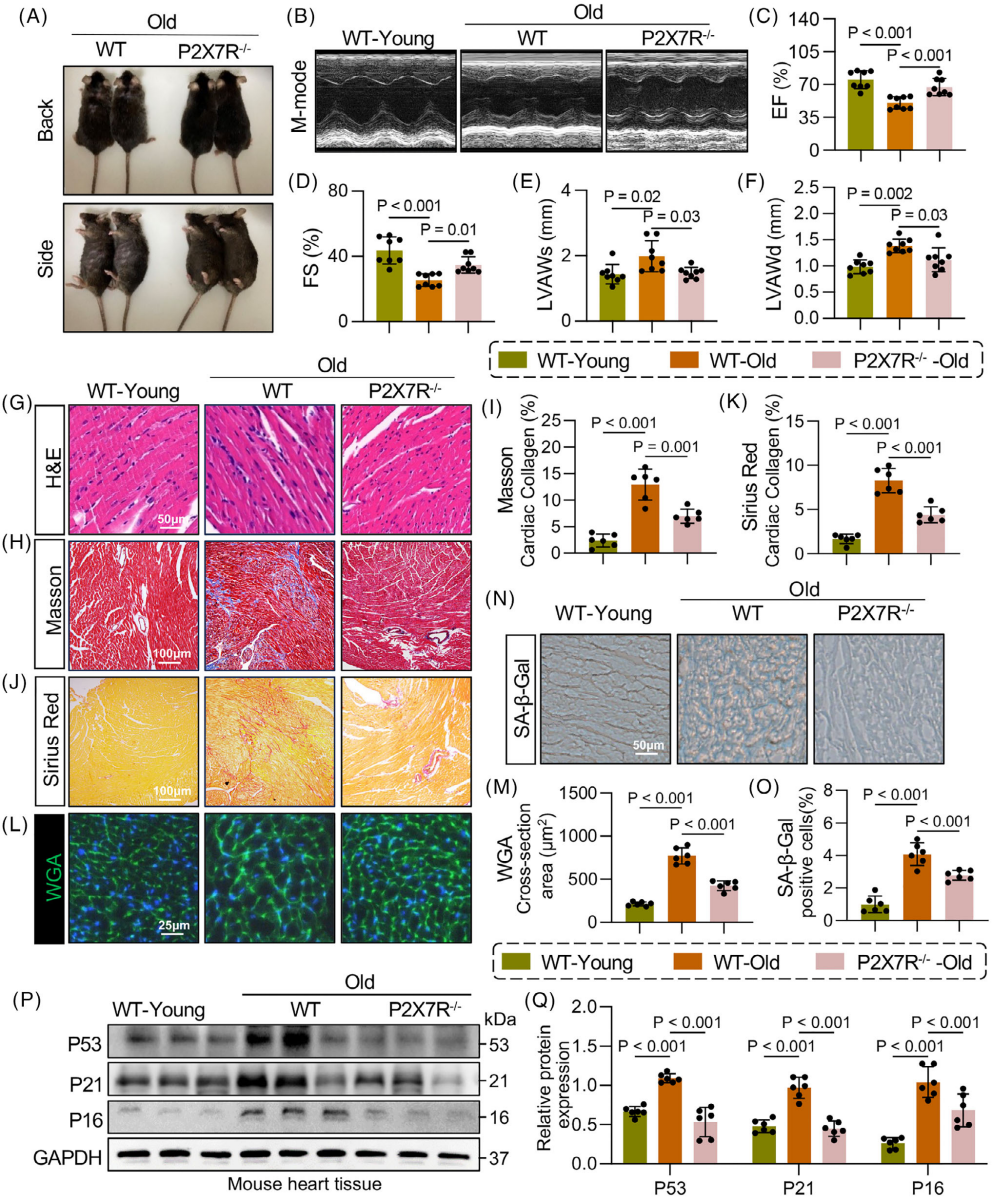
Purinergic 2×7 receptor (P2X7R) deficiency delays ageing in mice and alleviates ageing‐induced cardiac remodelling. (A) Representative image of wild type (WT)‐old and P2X7R^−/−^‐old mice. (B) Representative M‐mode echocardiographic images of left ventricles (LVs) from WT‐young, WT‐old and P2X7R^−/−^‐old mice (*n* = 8). (C and D) LV ejection fraction (EF) and fractional shortening (FS) were assessed by echocardiography (*n* = 8). (E and F) LV anterior wall thickness (left ventricular anterior wall in systole [LVAWs] and left ventricular anterior wall in diastole [LVAWd]) was assessed by echocardiography (*n* = 8). (G) Representative images of haematoxylin and eosin (H&E) staining (scale bar, 50 µm). (H and I) Representative images of Masson staining (H) (scale bar, 100 µm) and quantification of the interstitial fibrotic area (I) (*n* = 6). (J and K) Representative images of Sirius Red (Sirius) staining (J) (scale bar, 100 µm) and quantification of the interstitial fibrotic area (K) (*n* = 6). (L and M) Representative images of wheat germ agglutinin (WGA)‐stained sections (L) (scale bar, 25 µm) and quantification of the cardiomyocyte cross‐sectional area (M) (*n* = 6). (N and O) Representative images of senescence‐associated β‐galactosidase (SA‐β‐Gal) staining of myocardial tissue (scale bar, 50 µm) and quantification of the percentage of the SA‐β‐Gal+ area (O) (*n* = 6). (P) Representative Western blot analysis of tumour protein 53 (P53), CDN1A (P21) and p16INK4a (P16) levels in the myocardial tissue of WT‐young, WT‐old and P2X7R^−/−^‐old mice. GAPDH was used as a loading control (*n* = 6). (Q) Densitometric quantification of the immunoblots in (P) (*n* = 6). Adjusted *p*‐values are provided in the case of multiple group comparisons. P2X7R^−/−^, mice with whole‐body P2X7R knockout. GAPDH, Glyceraldehyde‐3‐Phosphate Dehydrogenase.

To investigate the potential role of P2X7R in cardiac ageing, we generated P2X7R‐KO (P2X7R^−/−^) mice and their WT littermates (Figure S2A,B). We observed that compared with age‐matched WT mice, young P2X7R‐KO mice did not significantly differ in terms of cardiac function (assessed by EF and FS), heart weight‐to‐tibia length ratio (HW/TL), cardiac hypertrophy (evaluated by left ventricular anterior wall in diastole [LVAWd] and left ventricular anterior wall in systole [LVAWs]), or myocardial fibrosis (determined by Masson and Sirius Red staining) (Figure S2C‒O). Male P2X7R^−/−^ mice and their WT littermates were subsequently maintained for 24 months (Figure S2P). We observed significant hair loss in the WT‐old mice and denser and smoother hair in the P2X7R^−/−^‐old mice (Figure 2A). Echocardiography was used to assess cardiac function in the mice (Figure 2B), revealing that compared with WT‐young mice, WT‐old mice exhibited significant cardiac dysfunction (Figure 2C,D) and cardiac hypertrophy (Figure 2E,F), whereas P2X7R deficiency markedly attenuated these age‐related cardiac impairments. Consistent with these findings, the HW/TL ratios followed a similar trend (Figure S2Q). Furthermore, histological analysis revealed abnormal cardiac structure (Figure 2G), significant cardiac fibrosis (Figure 2H‒K), and an increased cross‐sectional area of cardiomyocytes (Figure 2L,M) in WT‐old mice compared with those in WT‐young mice. Notably, these age‐associated cardiac abnormalities were markedly ameliorated in P2X7R^−/−^ mice. These results suggest that P2X7R deficiency significantly attenuates cardiac dysfunction and cardiac remodelling in aged mice. Additionally, P2X7R deficiency effectively reduced the expression of senescence proteins in cardiac tissue, including p21, p53 and p16 (Figure 2P,Q), and SA‐β‐Gal staining showed a consistent trend (Figure 2N,O). Together, these findings indicate that P2X7R deficiency delays ageing in mice and ameliorates ageing‐induced cardiac abnormalities.

### 3.3 P2X7R mediates ROS production and mitochondrial dysfunction in the ageing heart

**FIGURE 3:**
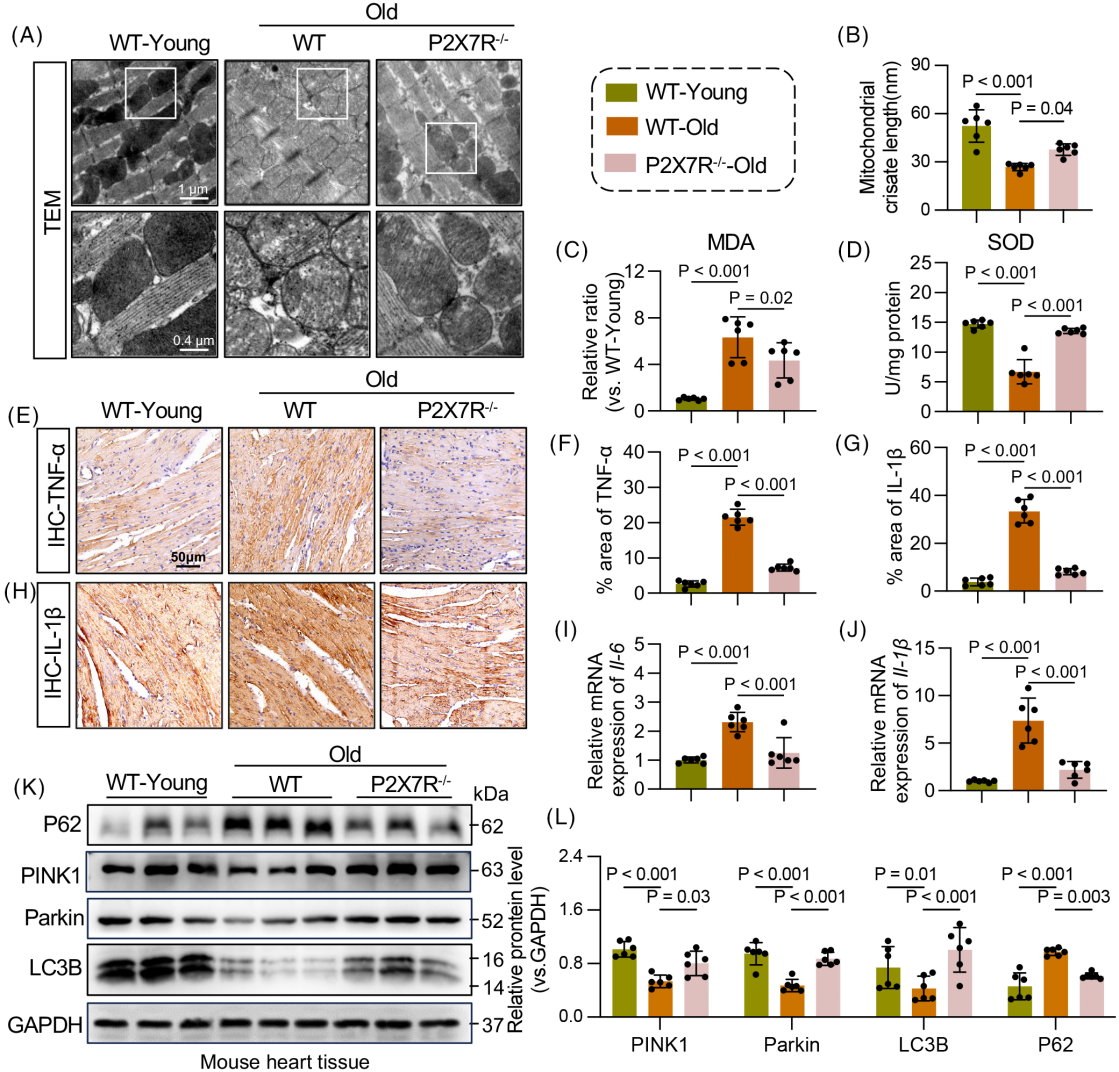
Purinergic 2×7 receptor (P2X7R) mediates reactive oxygen species (ROS) production and mitochondrial dysfunction during heart ageing. (A) Ultrastructural changes, including decreased mitochondrial volume, increased bilayer membrane density and the disappearance of mitochondrial cristae, were detected by transmission electron microscopy (TEM). (B) Mitochondrial crista length determined by TEM (*n* = 6). (C) Serum levels of malondialdehyde (MDA) in wild type (WT)‐young, WT‐old and P2X7R^−/−^‐old mice (*n* = 6). (D) Superoxide dismutase (SOD) activity in the myocardial tissue of WT‐young, WT‐old and P2X7R^−/−^‐old mice (*n* = 6). (E‒H) Representative images of tumour necrosis factor‐alpha (TNF‐α) (E) and interleukin‐1β (IL‐1β) (H) immunoreactivity in the myocardium of WT‐young, WT‐old and P2X7R^−/−^‐old mice (scale bar, 50 µm) and quantitative analysis of the TNF‐α (F) and IL‐1β (G) area ratios (*n* = 6). (I and J) mRNA levels of IL‐6 (I) and IL‐1β (J) in myocardial tissue (*n* = 6). (K) Representative Western blot analysis of sequestosome‐1, SQSTM1 (P62), PTEN‐induced putative kinase 1 (PINK1), PARK2 (Parkin) and LC3B (microtubule‐associated protein 1 light chain 3B) levels in the myocardial tissue of WT‐young, WT‐old and P2X7R^−/−^‐old mice. GAPDH was used as a loading control (*n* = 6). (L) Densitometric quantification of the immunoblots in (K) (*n* = 6). Adjusted *p*‐values are provided in the case of multiple group comparisons. P2X7R^−/−^, mice with whole‐body P2X7R knockout. GAPDH, Glyceraldehyde‐3‐Phosphate Dehydrogenase.

Recent findings have indicated that mitochondrial abnormalities represent a critical pathological hallmark of ageing. With advancing age, the activation of certain pathways, such as PTEN‐induced putative kinase 1 (PINK1)‒PARK2 (Parkin), markedly decreases, leading to impaired mitophagy signalling. This deficiency results in the accumulation of damaged mitochondria, which release excessive amounts of ROS and promote senescence.^19^ To investigate whether P2X7R modulates ageing‐induced cardiac remodelling through cardiac mitophagy, we used transmission electron microscopy to assess mitochondrial structure. In WT‐old mice, mitochondria in cardiac tissue exhibited marked swelling and loss of cristae, indicating mitochondrial dysfunction; however, P2X7R deficiency effectively reversed these mitochondrial ultrastructural abnormalities (Figure 3A,B). We further assessed the malondialdehyde (MDA) content and superoxide dismutase (SOD) activity to evaluate the ROS status and revealed significantly higher serum MDA levels and lower SOD activity in the cardiac tissue of WT‐old mice than in those of WT‐young mice; these alterations were blunted by P2X7R deficiency (Figure 3C,D). In addition, we assessed mitochondrial function and ROS levels in HL‐1 cardiomyocytes and revealed that P2X7R inhibition attenuated D‐gal‐induced mitochondrial dysfunction and ROS accumulation (Figure S3A‒D), which is consistent with the trends observed in mice.

As previously reported, inflammatory factors and mitophagy are interconnected drivers of cellular ageing. In brief, senescent cells secrete large amounts of proinflammatory cytokines, such as interleukin‐1β (IL‐1β), IL‐6 and tumour necrosis factor‐alpha (TNF‐α), leading to the development of a senescence‐associated secretory phenotype (SASP). SASP stimulates ROS production, which exacerbates mitochondrial damage and further amplifies inflammatory cytokine release and SASP formation.^20^ Here, the levels of proinflammatory factors such as TNF‐ɑ, IL‐6 and IL‐1β were significantly elevated in the heart tissues of WT‐old mice, but these increases were abolished in P2X7R^−/−^‐old mice (Figure 3E‒J). We subsequently assessed the levels of key proteins involved in mitophagy and found that compared with those in WT‐young mice, cardiac PINK/Parkin pathway activity and LC3‐II (microtubule‐associated protein 1 light chain 3) expression were significantly decreased and sequestosome‐1, SQSTM1 (P62) expression was elevated in the heart tissues of WT‐old mice, suggesting impaired cardiac mitophagy and a consequent failure to efficiently clear damaged mitochondria. Notably, these ageing‐related deficits in mitophagy were attenuated by P2X7R deficiency (Figure 3K,L). Additionally, we observed the same trend in vitro (Figure S3D‒F). Collectively, our findings suggest that P2X7R depletion enhances mitophagy during cardiac ageing.

### 3.4 P2X7R deficiency increases NR4A1 expression to alleviate ageing‐induced cardiac mitophagy

**FIGURE 4:**
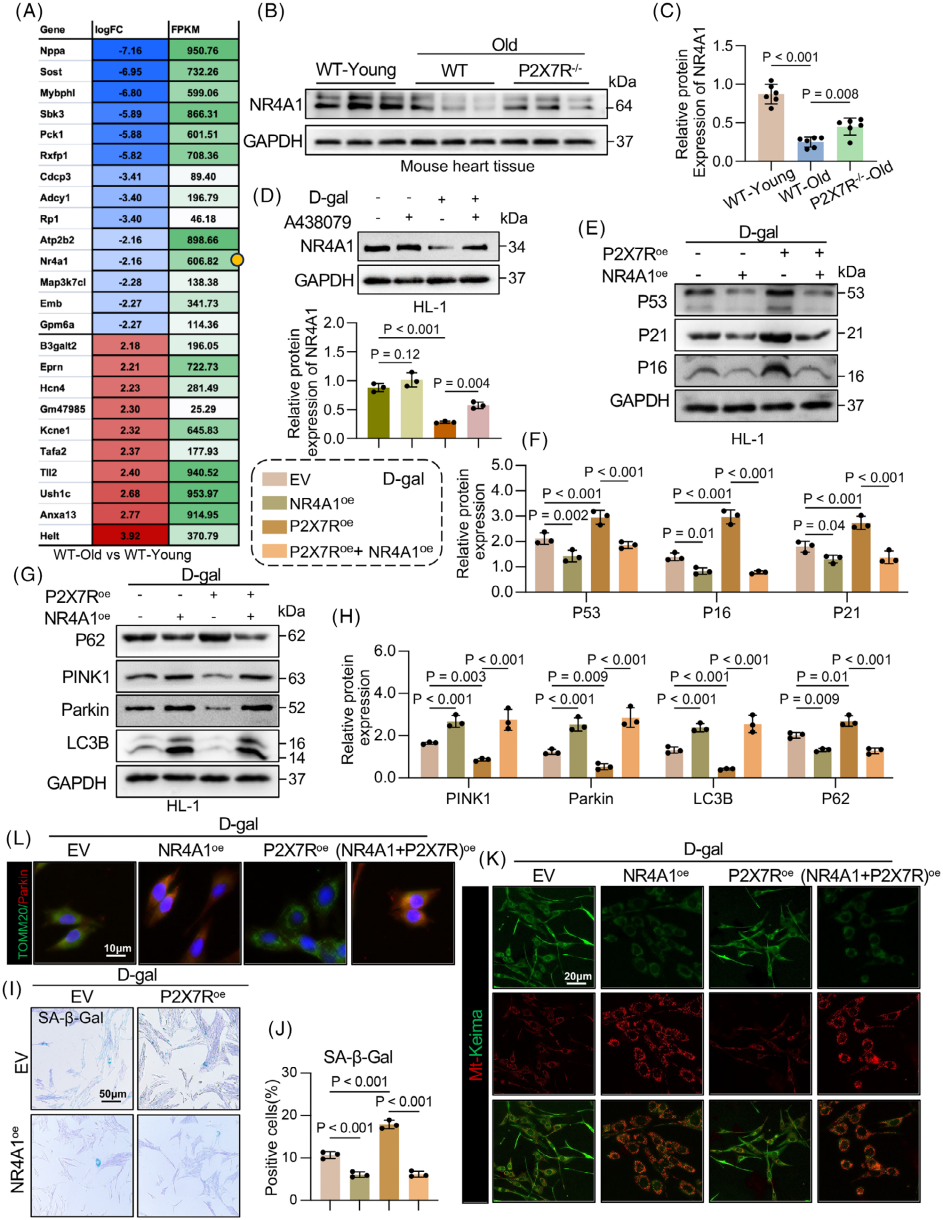
Purinergic 2×7 receptor (P2X7R) deficiency increases nuclear receptor subfamily 4 group A member 1 (NR4A1) expression to alleviate ageing‐induced cardiac mitophagy. (A) Heatmap showing differentially expressed genes in wild type (WT)‐old and P2X7R^−/−^‐old mouse hearts (*n* = 3). (B and C) Representative Western blot analysis of NR4A1 expression in the myocardial tissue of WT‐young, WT‐old and P2X7R^−/−^‐old mice; GAPDH was used as a loading control (B) and density analysis (C) (*n* = 6). (D) NR4A1 expression in the Ctrl, A438079, D‐galactose (D‐gal) and A438079 + D‐gal groups and density analysis (*n* = 3). (E and F) Representative Western blot analysis of tumour protein 53 (P53), CDN1A (P21) and p16INK4a (P16) levels in D‐gal‐induced HL‐1 cells treated with P2X7R overexpression (P2X7R^oe^) or NR4A1 overexpression (NR4A1^oe^); GAPDH was used as a loading control (E) and densitometric quantification (F) (*n* = 3). (G and H) Representative Western blot analysis of sequestosome‐1, SQSTM1 (P62), PTEN‐induced putative kinase 1 (PINK1), PARK2 (Parkin) and LC3B (microtubule‐associated protein 1 light chain 3B) levels in D‐gal‐induced HL‐1 cells treated with P2X7R^oe^ or NR4A1^oe^ (*n* = 3). GAPDH was used as a loading control (G) and density analysis (H). GAPDH was used as a loading control. (I) Representative images of SA‐β‐Gal staining of HL‐1 cells (*n* = 3). (J) The percentages of SA‐β‐Gal+ cells in (I) were quantified (*n* = 3). (K) Representative confocal images of mt‐Keima expression in each group (*n* = 3). (L) Double immunofluorescence staining for Parkin (red) and TOMM20 (green) in HL‐1 cells treated with D‐gal, NR4A1^oe^ or P2X7R^oe^ (*n* = 3). Merged images (orange) show colocalisation (scale bar, 20 µm). Adjusted *p*‐values are provided in the case of multiple group comparisons. P2X7R^−/−^, mice with whole‐body P2X7R knockout. GAPDH, Glyceraldehyde‐3‐Phosphate Dehydrogenase.

To investigate the potential molecular mechanisms underlying P2X7R‐mediated cardiac ageing and impaired mitophagy, we performed a cardiac transcriptome sequencing analysis and compared the WT‐old group with the WT‐young group. Among the most significantly differentially expressed genes (Figure 4A), nuclear receptor subfamily 4 group A member 1 (NR4A1) emerged as a key candidate because of its established role in regulating ageing and mitophagy.^17^, ^18^ Furthermore, we assessed NR4A1 protein expression in mouse heart tissues (Figure 4B,C) and D‐gal‐stimulated HL‐1 cardiomyocytes (Figure 4D) and found a significant decrease in NR4A1 levels during cardiac ageing or cardiomyocyte senescence. Notably, P2X7R deficiency or inhibition reversed this decline. Thus, we speculated that NR4A1 may function as a downstream effector of P2X7R in regulating mitophagy in ageing cardiomyocytes. To validate this hypothesis, we inhibited NR4A1 activity using a selective NR4A1 inhibitor, DIM8, and overexpressed NR4A1 in HL‐1 cardiomyocytes via plasmid transfection (Figure S4A). Further assessment of cardiomyocyte ageing and mitophagy revealed that NR4A1 inhibition significantly exacerbated the D‐gal‐induced senescence phenotype and impaired mitophagy (Figure S5A‒H), whereas NR4A1 overexpression markedly ameliorated the D‐gal‐induced senescence phenotype and restored mitophagy function (Figure S5I‒P). These results confirm that NR4A1 functions as a protective regulator of cardiomyocyte senescence. We subsequently constructed plasmids overexpressing P2X7R (Figure S4B) or NR4A1 to cotransfect HL‐1 cells following D‐gal stimulation. We found that P2X7R overexpression significantly exacerbated the D‐gal‐induced senescence phenotype and impaired mitophagy, effects that were reversed by NR4A1 overexpression (Figure 4E‒L). These findings indicate that P2X7R promotes cardiomyocyte senescence by inhibiting mitophagy through NR4A1.

**FIGURE 5:**
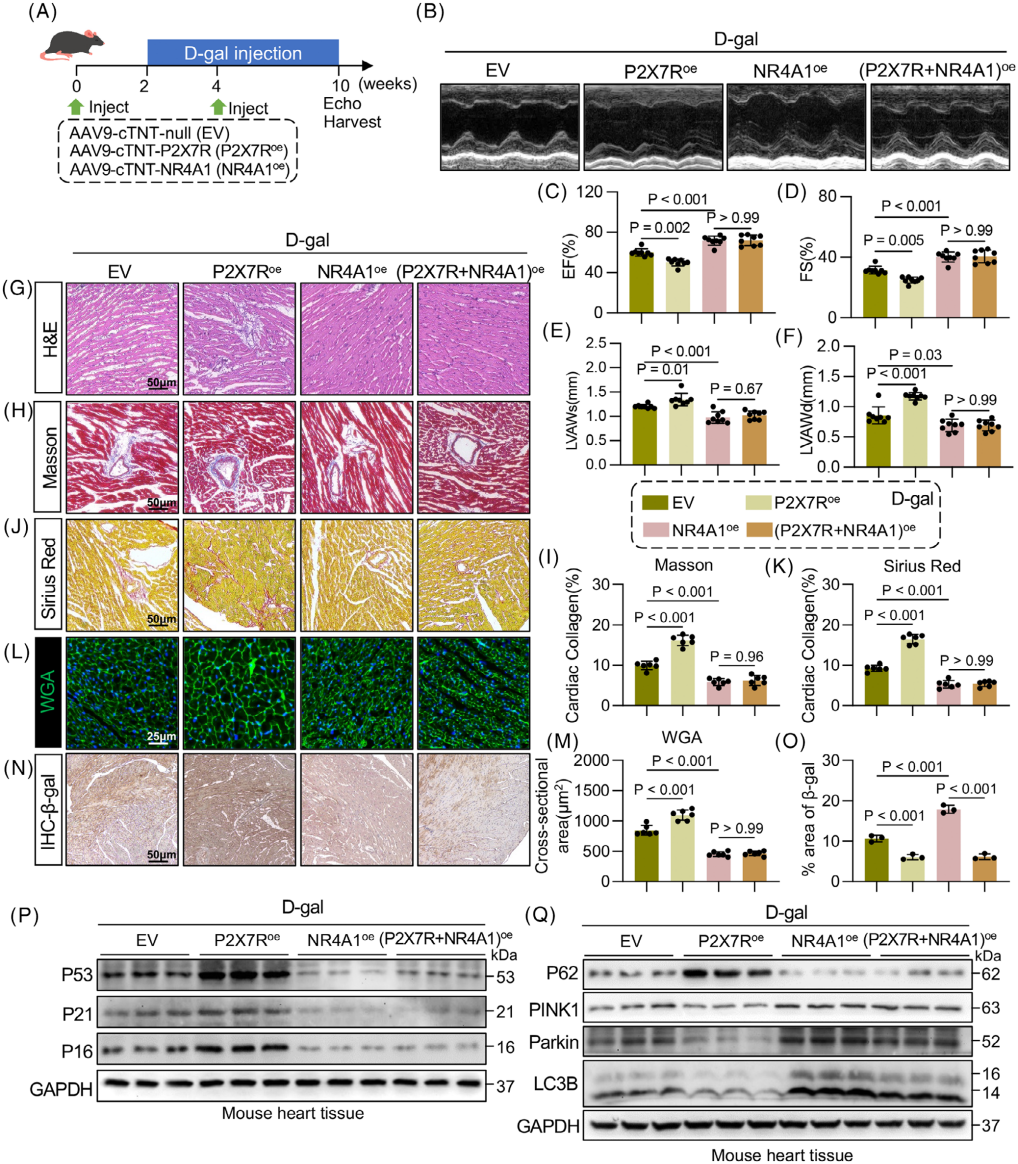
Overexpression of nuclear receptor subfamily 4 group A member 1 (NR4A1) eliminates Purinergic 2×7 receptor (P2X7R)‐mediated cardiac remodelling and mitophagy in D‐galactose (D‐gal)‐induced mice. (A) Schematic diagram depicting the experimental strategy for the D‐gal‐induced ageing model and the activation of NR4A1 and P2X7R. (B) Representative M‐mode echocardiographic images of the left ventricle from D‐gal + EV, D‐gal + P2X7R^oe^, D‐gal + NR4A1^oe^ or D‐gal + P2X7R^oe^ + NR4A1^oe^ mice (*n* = 8). (C and D) LV ejection fraction and fractional shortening were assessed by echocardiography (*n* = 8). (E and F) Left ventricular anterior wall thickness (left ventricular anterior wall in systole [LVAWs] and left ventricular anterior wall in diastole [LVAWd]) was assessed by echocardiography (*n* = 8). (G) Representative images of haematoxylin and eosin (H&E) staining (scale bar, 50 µm). (H and I) Representative images of Masson staining (H) (scale bar, 50 µm) and quantification of the interstitial fibrotic area (I) (*n* = 6). (J and K) Representative images of Sirius Red staining (J) (scale bar, 50 µm) and quantification of the interstitial fibrotic area (K) (*n* = 6). (L and M) Representative images of wheat germ agglutinin (WGA)‐stained sections (L) (scale bar, 50 µm) and quantification of cardiomyocyte cross‐sections (M) (*n* = 6). (N and O) Representative images of β‐Gal immunoreactivity in myocardial tissue (scale bar, 50 µm) and quantification of the percentage of the β‐Gal^+^ area (O) (*n* = 6). (P) Representative Western blot analysis of tumour protein 53 (P53), CDN1A (P21) and p16INK4a (P16) levels in the myocardial tissue of D‐gal + EV, D‐gal + P2X7R^oe^, D‐gal + NR4A1^oe^ and D‐gal + P2X7R^oe^ + NR4A1^oe^ mice. GAPDH was used as a loading control (*n* = 6). (Q) Representative Western blot analysis of sequestosome‐1, SQSTM1 (P62), PTEN‐induced putative kinase 1 (PINK1), PARK2 (Parkin) and LC3B (microtubule‐associated protein 1 light chain 3B) levels in the myocardial tissue of D‐gal + EV, D‐gal + P2X7R^oe^, D‐gal + NR4A1^oe^ and D‐gal + P2X7R^oe^ + NR4A1^oe^ mice. GAPDH was used as a loading control (*n* = 6). Adjusted *p*‐values are provided in the case of multiple group comparisons. P2X7R^oe^, AAV9‐cTnT‐P2X7R; NR4A1^oe^, AAV9‐cTnT‐NR4A1; EV, AAV9‐cTnT‐EV. GAPDH, Glyceraldehyde‐3‐Phosphate Dehydrogenase.

Furthermore, to elucidate the role of the cardiac‐specific P2X7R‒NR4A1 axis in cardiac ageing and mitophagy, we overexpressed P2X7R or NR4A1 specifically in cardiomyocytes with AAV9 (AAV9‐cTnT‐P2X7R^oe^ or AAV9‐cTnT‐NR4A1^oe^) (Figure S6A‒C). Moreover, we established disease‐related mouse models of senescence, including Ang II‐induced hypertension, diabetic cardiomyopathy‐related heart failure and D‐gal‐induced senescence, and assessed cardiac senescence in these three models. We found that compared with the other two disease‐related models, D‐gal‐treated mice exhibited a more pronounced senescence phenotype (Figure S6D‒F). Therefore, we selected the D‐gal‐induced senescence mouse model for subsequent in vivo experiments. AAV9‐cTnT‐P2X7R^oe^ or AAV9‐cTnT‐NR4A1^oe^ was administered to WT mice for 2 weeks to induce exogenous cardiomyocyte‐specific overexpression of P2X7R or NR4A1 prior to 6 weeks of D‐gal administration (Figure 5A). We observed markedly coarse and sparse hair in AAV9‐cTnT‐P2X7R^oe^ + D‐gal mice, whereas the hair appeared denser and smoother in mice administered AAV9‐cTnT‐NR4A1^oe^ (Figure S6G). Echocardiography was performed to evaluate cardiac function in mice from each group (Figure 5B), which revealed that the cardiomyocyte‐specific overexpression of P2X7R significantly exacerbated cardiac dysfunction and cardiac hypertrophy, whereas these adverse effects were markedly attenuated by the cardiomyocyte‐specific overexpression of NR4A1 (Figure 5C‒F). Consistent with these findings, the HW/TL ratios, cardiac structure, cardiac fibrosis, cardiomyocyte cross‐sectional area, cardiac senescence and mitophagy exhibited similar trends (Figures 5G‒Q and S6H‒J). Collectively, these results indicate that NR4A1 functions as a downstream effector through which cardiomyocyte‐specific P2X7R promotes cardiac senescence.

### 3.5 TRIM26 directly binds NR4A1 and promotes its degradation

**FIGURE 6:**
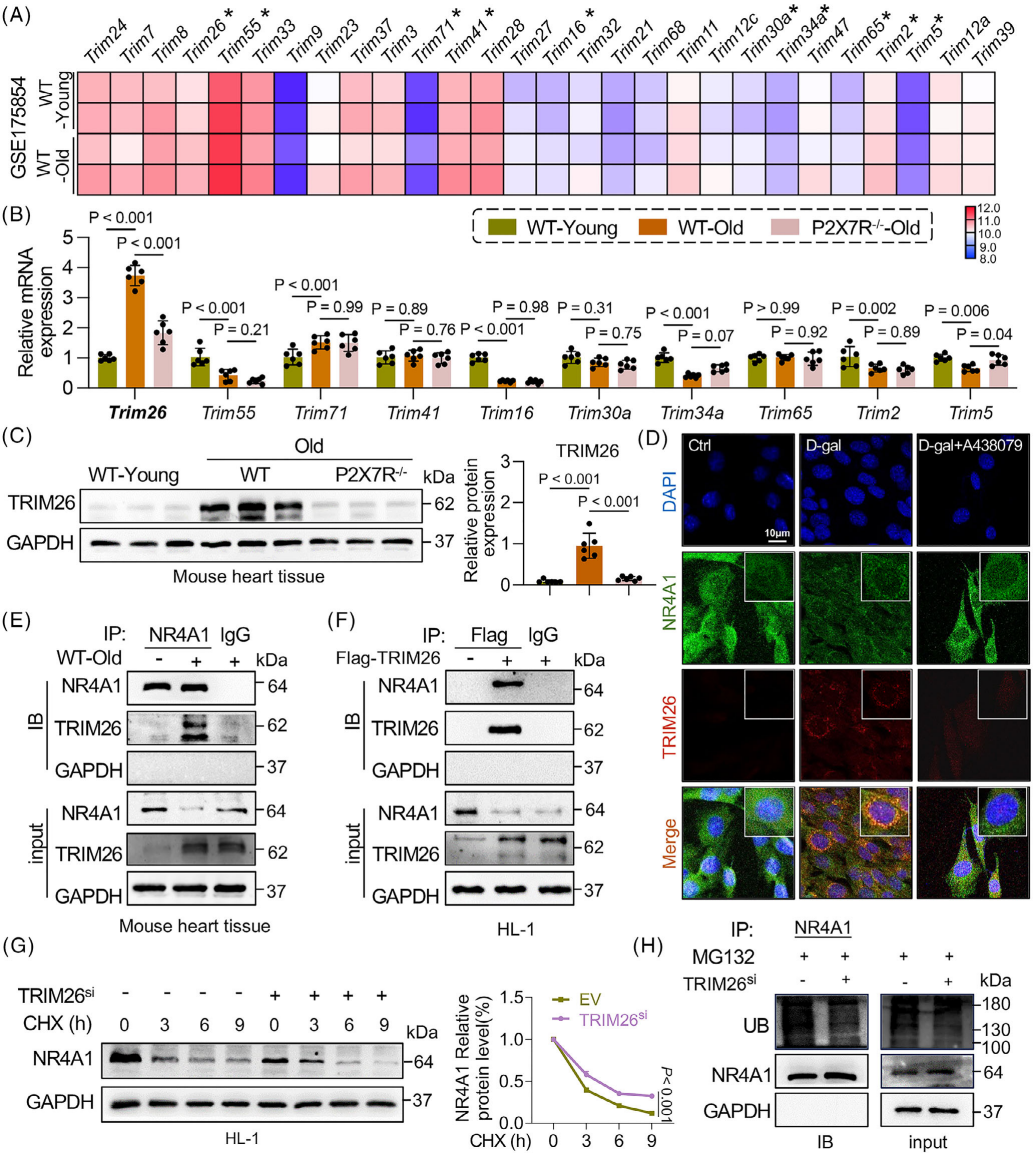
Tripartite motif containing 26 (TRIM26) directly binds to nuclear receptor subfamily 4 group A member 1 (NR4A1) and promotes its degradation. (A) Heatmap of TRIM family expression profiles in mouse models following ageing from the GSE175854 dataset (*n* = 2). (B) mRNA levels of TRIM26, TRIM55, TRIM71, TRIM41, TRIM16, TRIM30a, TRIM34a, TRIM65, TRIM2 and TRIM5 in the myocardial tissue of wild type (WT)‐young, WT‐old and P2X7R^−/−^‐old mice (*n* = 6). (C) TRIM26 expression in the myocardial tissue of WT‐young, WT‐old and P2X7R^−/−^‐old mice; GAPDH was used as a loading control; and density analysis (*n* = 6). (D) Double immunofluorescence staining for TRIM26 (red) and NR4A1 (green) in HL‐1 cells treated with D‐galactose (D‐gal) or A438079. Merged images (orange) show colocalisation (scale bar, 10 µm). (E and F) Coimmunoprecipitation (Co‐IP) experiments of TRIM26 and NR4A1 in myocardial tissue (E) and HL‐1 cells (F). (G) Protein level of NR4A1 in HL‐1 cells after treatment with cycloheximide (CHX) and density analysis (*n* = 3). (H) Ubiquitination level of NR4A1 under TRIM26 silencing (TRIM26^si^) plasmid treatment. Adjusted *p*‐values are provided in the case of multiple group comparisons. P2X7R^−/−^, mice with whole‐body P2X7R knockout; TRIM26^oe^, TRIM26 overexpression. GAPDH, Glyceraldehyde‐3‐Phosphate Dehydrogenase.

To further elucidate the regulatory mechanism of P2X7R on NR4A1, we examined the mRNA expression level of NR4A1. The results revealed that the decrease in its protein level was significantly greater than the change in its mRNA level (Figure S7A), suggesting that this regulation may involve posttranslational modifications. Among the known posttranslational modification pathways closely associated with protein expression, the ubiquitin–proteasome system represents a key mechanism,^21^ which prompted us to further investigate whether this pathway is involved. Numerous studies have shown that NR4A1 expression can be regulated by ubiquitination.^22^ E3 ligases, which are common ubiquitin‐protein ligases, have attracted interest in cardiovascular disorders. To determine the role of E3 ligases in cardiac senescence, a comprehensive search of TRIM family expression in a GEO dataset (GSE175854, comprising samples from the myocardial tissue of normal young mice and old mice) was conducted. The expression of 10 members of the TRIM family (TRIM26, TRIM55, TRIM71, TRIM41, TRIM16, TRIM30a, TRIM34a, TRIM65, TRIM2 and TRIM5) differed between young mice and old mice (Figure 6A). We then evaluated the mRNA levels of these 10 TRIM family members in cardiac tissues from WT‐young, WT‐old and P2X7R^−/−^‐old mice. Notably, only TRIM26 mRNA was significantly upregulated in aged WT hearts but decreased in aged P2X7R^−/−^ hearts (Figure 6B). Additionally, the TRIM26 protein level showed a similar trend in both cardiac tissue (Figure 6C) and HL‐1 cells in vitro (Figure S7B), indicating that TRIM26 expression is regulated by P2X7R during ageing. Therefore, we hypothesise that TRIM26 may regulate NR4A1 expression in the context of P2X7R‐mediated cardiac ageing. Double‐immunofluorescence staining revealed increased colocalisation of TRIM26 and NR4A1 in HL‐1 cardiomyocytes after D‐gal treatment (Figure 6D), suggesting a potential direct interaction between TRIM26 and NR4A1. To validate this association, coimmunoprecipitation assays were conducted using cardiac tissues from aged mice (Figure 6E) as well as in HL‐1 cells transfected with a TRIM26 plasmid (Figure 6F), and it was confirmed that TRIM26 physically interacts with NR4A1.

To elucidate how TRIM26 regulates NR4A1 expression and whether this regulation involves NR4A1 ubiquitination, we silenced TRIM26 using siRNA (Figure S7C) and overexpressed TRIM26 via plasmid transfection (Figure S7D) in HL‐1 cardiomyocytes. Following TRIM26 silencing or overexpression, NR4A1 protein levels decreased or increased, respectively (Figure S7E,F). Next, we transfected cells with TRIM26‐targeting siRNA, treated them with cycloheximide to inhibit protein synthesis, and measured NR4A1 protein levels at different time points (0, 3, 6 and 9 h). As shown in Figure 6G, following cycloheximide treatment, NR4A1 protein degradation was significantly slower in the TRIM26^si^ group than in the control group, indicating that TRIM26 promotes NR4A1 protein degradation in cardiomyocytes. To elucidate the mechanism by which TRIM26 acts on the NR4A1 protein, we overexpressed TRIM26 in HL‐1 cells in the presence of the proteasome inhibitor MG132 or one of three autophagy inhibitors (3‐methyladenine, chloroquine or bafilomycin A1). We found that MG132 effectively reversed TRIM26‐mediated NR4A1 degradation, whereas the autophagy inhibitors had no effect, indicating that NR4A1 protein degradation occurs via the proteasomal pathway (Figure S7G). In addition, the level of ubiquitinated NR4A1 was significantly lower in the TRIM26‐silenced cells than in the control cells (Figure 6H). Together, these results show that TRIM26 directly binds to NR4A1 and promotes its degradation through ubiquitination.

### 3.6 P2X7R stabilises TRIM26 mRNA and decreases its degradation in ageing hearts via HuR

**FIGURE 7:**
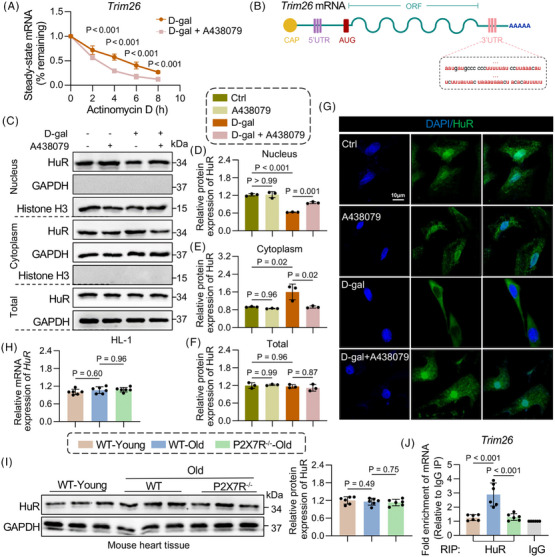
Purinergic 2×7 receptor (P2X7R) decreased tripartite motif containing 26 (TRIM26) mRNA degradation in ageing hearts by affecting human antigen R (HuR). (A) D‐galactose (D‐gal)‐induced HL‐1 cells were pretreated with DMSO or A438079, treated with actinomycin D and monitored for 2, 4, 6 and 8 h, after which the mRNA expression of TRIM26 was measured by RT‒qPCR (*n* = 3). (B) Schematic representation of the TRIM26 mRNA structure. (C) HuR expression in the nuclei of HL‐1 cells treated with D‐gal or A438079. Histone H3 was used as a loading control for the nuclear expression of HuR, and GAPDH was used as a loading control for the cytoplasmic and total expression of HuR (*n* = 3). (D–F) Densitometric quantification of the immunoblots in (C). (G) Immunofluorescence staining for HuR (green) in HL‐1 cells treated with D‐gal or A438079 (scale bar, 50 µm). (H and I) Protein and mRNA expression of HuR in the myocardial tissue of wild type (WT)‐young, WT‐old and P2X7R^−/−^‐old mice and density analysis (*n* = 6). (J) Myocardial tissue of WT‐old, WT‐old and P2X7R^−/−^‐old mice was subjected to RNA immunoprecipitation for TRIM26 mRNA using an anti‐HuR antibody (*n* = 6). Adjusted *p*‐values are provided in the case of multiple group comparisons. P2X7R^−/−^, mice with whole‐body P2X7R knockout. DMSO, Dimethyl Sulfoxide; GAPDH, Glyceraldehyde‐3‐Phosphate Dehydrogenase.

The above findings suggest that P2X7R regulates TRIM26 expression at both the mRNA and protein levels. We speculated that the decrease in TRIM26 expression caused by P2X7R loss may result from either decreased synthesis or increased mRNA degradation. To investigate this, we first examined TRIM26 mRNA stability in D‐gal‐stimulated HL‐1 cells with or without the P2X7R antagonist A438079. The half‐life of TRIM26 mRNA was significantly shorter in the A438079 + D‐gal group than in the D‐gal alone group, indicating that A438079 accelerated TRIM26 mRNA degradation and that P2X7R affects the stability of TRIM26 mRNA (Figure 7A). As previously reported, RNA‐binding proteins such as HuR are regulators of posttranscriptional gene expression, typically through binding to AU‐rich elements (AREs) or U‐rich motifs in the 3′ untranslated regions (UTRs) of target mRNAs. Additionally, our previous study demonstrated that P2X7R deficiency modulates HuR expression in the context of Ang II‐induced cardiac remodelling.^14^ Therefore, we evaluated whether the regulation of TRIM26 by P2X7R is mediated by HuR. As expected, multiple AREs or U‐rich motifs are present in the 3′UTR of TRIM 26 mRNA (Figure 7B), which are potential binding sites for HuR. Interestingly, D‐gal treatment promoted nuclear‐to‐cytoplasmic shuttling of HuR, increased its binding to TRIM26 mRNA, and thereby stabilised and upregulated TRIM26 expression. However, these effects were markedly abrogated by A438079 (Figure 7C‒G). Notably, total HuR levels remained unchanged both in vitro (Figure 7C,F) and in vivo (Figure 7H,I). Finally, an RNA immunoprecipitation (RIP) assay was performed to validate the binding capacity of HuR to TRIM26 mRNA. As shown in Figure 7J, HuR directly bound to TRIM26 in the heart tissues of WT‐old mice, and this interaction was significantly reduced upon P2X7R deficiency. These results indicate that P2X7R specifically induces HuR nuclear shuttling, thereby increasing the binding of HuR to TRIM26 mRNA and ultimately leading to altered TRIM26 expression in cardiomyocytes.

## 4 DISCUSSION

Ageing serves as an independent risk factor for various disorders, including cardiac dysfunction and vascular remodelling^23^, ^24^, ^25^; however, the underlying mechanism remains largely unclear. Cardiac mitophagy is essential for removing damaged mitochondria and maintaining cardiac function during ageing. In this study, we identified a previously unrecognised role of P2X7R, which is positively correlated with ageing in humans and cardiac ageing in mice. First, we found that serum P2X7R levels increased progressively with age in humans, whereas elevated cardiac P2X7R expression in aged mice coincided with decreased cardiac function and impaired mitophagy. Second, P2X7R deficiency attenuated ageing‐induced cardiac remodelling and dysfunction, ameliorated the senescence features of aged hearts and restored defective mitophagy. Conversely, cardiomyocyte‐specific P2X7R overexpression exacerbated D‐gal‐induced cardiac dysfunction, accelerated senescence, and further disrupted mitophagy induced by D‐gal. Third, we demonstrated that P2X7R regulates cardiomyocyte mitophagy by promoting the nucleocytoplasmic shuttling of HuR, which increases TRIM26 mRNA stability and facilitates the ubiquitination‐mediated degradation of NR4A1, ultimately suppressing mitophagy. Collectively, our findings indicate that P2X7R acts as a key contributor to cardiac ageing by modulating the HuR/TRIM26/NR4A1 axis and impairing mitophagy.

P2X7R is highly expressed in immune cells, cardiomyocytes, fibroblasts and endothelial cells, where it mediates inflammatory responses by activating the NLRP3 inflammasome and promoting the release of proinflammatory cytokines such as IL‐1β and IL‐18.^12^, ^26^ These cytokines contribute to the progression of atherosclerosis, myocardial infarction and arrhythmias. Recently, P2X7R deficiency or pharmacological inhibition has been shown to mitigate cardiac fibrosis by regulating cardiomyocyte apoptosis or ferroptosis in models of diabetic cardiomyopathy^27^ and hypertensive heart disease.^14^ Given its substantial role, P2X7R is emerging as a potential therapeutic target for cardiovascular diseases. However, the aforementioned studies were performed primarily in young animals. In the present study, we provide evidence that serum P2X7R levels are positively associated with ageing in humans. In aged mice, P2X7R deficiency or pharmacological inhibition reduced cardiomyocyte senescence and exacerbated ageing‐induced cardiac remodelling, ultimately improving cardiac function; these findings suggest that P2X7R is a key regulatory factor in cardiac ageing. Therefore, exogenous supplementation with a P2X7R inhibitor may offer potential health benefits in older individuals. While our data suggest a correlation between circulating and cardiac P2X7R levels, serum measurements remain indirect and may be influenced by systemic sources. Direct validation in human cardiac tissues across ages is therefore warranted. Furthermore, although our systemic P2X7R‐KO model provides important insights, the observed benefits on cardiac ageing could involve contributions from noncardiomyocyte populations. Future studies employing cardiomyocyte‐specific conditional knockout models will be crucial to definitively establish the cell‐autonomous role of P2X7R signalling in the ageing heart.

Mechanistically, we identified the nuclear receptor NR4A1 as a downstream regulatory factor of P2X7R that regulates mitophagy in cardiomyocytes. NR4A1, also known as Nur77,^28^ can be ubiquitinated and bind to p62 to mediate celastrol‐induced mitophagy^29^ and can also interact with TNF receptor‐associated factor 2 to drive mitophagy, thereby alleviating inflammation.^30^, ^31^ Considering that appropriately increased mitophagy favours the alleviation of myocardial ageing, we speculated that NR4A1 overexpression is closely associated with the regulation of cardiac ageing. Wang and coworkers reported that Nur77 mitigated age‐related cardiac fibrosis by directly initiating GSK‐3β.^17^ In the present study, we found that NR4A1 expression was notably inhibited in the hearts of aged mice, an effect that was reversed by P2X7R‐KO. We revealed that NR4A1 inhibition aggravated and that NR4A1 overexpression abolished cellular ageing. Moreover, NR4A1 overexpression counteracted the cellular ageing and mitochondrial autophagy caused by P2X7R overexpression. Thus, we provide favorable evidence that P2X7R promotes cardiac senescence by downregulating NR4A1 expression.

Although the NR4A1 protein level in the hearts of older mice is lower than that in young mice and increases after P2X7R depletion, the mechanism by which P2X7R regulates NR4A1 degradation remains unclear. Several studies have shown that P2X7R is important for NR4A1 activity and function through posttranslational modifications such as SUMOylation,^32^ phosphorylation^33^ and ubiquitination.^32^ Interestingly, tripartite motif (TRIM) family proteins, most of which have E3 ubiquitin ligase activity, are involved in diverse cellular processes, including autophagy,^34^ intracellular signalling,^35^ cardiac disease and tumourigenesis.^36^ TRIM proteins also interact with transcription factors such as NR4A1 or SOX2 to modulate their stability and function via ubiquitination. For example, TRIM13 interacts with NR4A1 and promotes its ubiquitination‐mediated degradation in breast cancer cells,^37^ whereas TRIM26 competes with WWP2 to control SOX protein levels in the context of glioblastoma.^38^ However, whether TRIM family proteins participate in cardiac senescence needs to be further clarified. In our study, we selected TRIM26 from the GEO database analysis and verified its expression. We found that TRIM26 protein expression was upregulated in aged hearts but reduced in aged hearts lacking P2X7R. Additionally, TRIM26 colocalises with and negatively regulates NR4A1 in cardiomyocytes. Furthermore, we confirmed that TRIM26 directly binds to NR4A1, which in turn promotes NR4A1 ubiquitination and its subsequent proteolysis. Although we established that P2X7R activation leads to the TRIM26‐mediated ubiquitination and proteasomal degradation of the NR4A1 protein, the mechanism underlying the concomitant reduction in NR4A1 mRNA levels observed in aged hearts remains unclear. Our transcriptomic analysis also revealed additional age‐associated alterations, such as the downregulation of Nppa, a classical biomarker of cardiac stress and a recently described extracellular regulator of autophagy, which may represent a parallel regulatory layer in cardiac ageing distinct from the NR4A1 pathway.

Notably, HuR, an RNA‐binding protein, binds to target mRNAs containing AU‐rich or U‐rich elements in their 3′UTR to regulate mRNA stability and turnover. Recent studies have indicated that the dysregulation of HuR is involved in various human diseases, including cancer^39^ and immune‐related disorders,^40^ and in drug resistance in different tumours.^41^ In previous research, we demonstrated that P2X7R modulates both the protein expression and the nucleocytoplasmic shuttling of HuR to regulate ferroptosis in Ang II‐induced cardiac remodelling.^14^ Here, ageing increased nuclear‐to‐cytoplasmic HuR shuttling in cardiomyocytes, a process that was attenuated by either P2X7R deletion or pharmacological inhibition. However, during this process, neither the protein nor the mRNA level of HuR significantly changed, indicating that P2X7R‐mediated cardiac ageing involves alterations in HuR nucleocytoplasmic shuttling only. The function of HuR is highly dependent on its shuttling between the nucleus and cytoplasm, and this process is highly dynamic and meticulously regulated by multiple signalling pathways involving multiple factors, such as the cell cycle, DNA damage,^42^ lncRNA interactions, posttranslational modifications and nuclear export proteins. While our study revealed a functional link between P2X7R activation and HuR translocation, the exact molecular mechanism remains to be fully determined. In addition, the abundant AU elements in the 3′UTR of TRIM26 mRNA provide the structural basis for its binding HuR, and the RIP results revealed an increase in the binding of HuR to TRIM26 mRNA in the hearts of aged mice, which subsequently increased the stability of TRIM26 and contributed to its upregulated expression. Thus, we provide the first evidence for the posttranscriptional regulation of TRIM26 expression by HuR in ageing hearts; our results strongly indicate that P2X7R mediates cardiac senescence by influencing the capacity of HuR to bind to TRIM26 mRNA. Furthermore, the pathway we propose is initiated by P2X7R, a cell‐surface receptor, and extends to downstream nuclear and cytoplasmic events. A key unresolved question is how P2X7R activation is linked to the nucleocytoplasmic shuttling of HuR. While we ruled out HuR phosphorylation as the key modification underlying its altered localisation (Figure S8A), the fundamental mechanism by which P2X7R activation triggers HuR nucleocytoplasmic shuttling remains unknown. Potential mechanisms may involve the interaction of HuR with nuclear transport proteins^34^ or the occurrence of other posttranslational modifications, such as methylation^43^ or indirect signalling cascades.^44^ Determining the specific mechanism that operates in this context will be an important direction for future research and will help complete the molecular map of this ageing‐related pathway.

Additionally, the exclusive use of male mice significantly limits the generalisability of our findings. Factors such as sex hormones, particularly estrogen and testosterone, are known to regulate cardiac inflammation,^45^, ^46^ mitochondrial function^47^ and the activity of nuclear receptors.^48^ It is therefore plausible that sex‐specific factors may influence P2X7R signalling, NR4A1 function or overall susceptibility to age‐related mitophagy decline. The development of testable hypotheses about these interactions can provide more in‐depth insights into potential sex differences in cardiac ageing mechanisms.

In summary, using P2X7R‐KO mice and a specific inhibitor, we elucidated that P2X7R mediates ageing‐induced cardiac dysfunction by impairing cardiomyocyte mitophagy. This study (1) provides direct evidence that serum P2X7R levels in humans are positively correlated with age; (2) reveals the previously unrecognised, essential role of P2X7R in mediating ageing‐induced cardiac alterations in mice; and (3) elucidates a new mechanism by which P2X7R promotes cardiac ageing through the HuR/TRIM26/NR4A1 axis to regulate ROS levels and cardiac mitophagy. These findings provide new insights into the molecular events underlying cardiac dysfunction in elderly mice and demonstrate that P2X7R overexpression exacerbates cardiac ageing, revealing that P2X7R is a potential novel therapeutic target for age‐related cardiac decline.

## AUTHOR CONTRIBUTIONS

Zhouqing Huang contributed to the literature search and study design. Yixin Zhou, Xin Zhong, Zhijie Mao, Yunxuan Chen, Jincheng Xing, Jiaxu Shen, Wenli Zhang, Ji Zhang, Jiaxuan Mei, Zhentong Yang, Zhuoqun Wang and Bozhi Ye performed the experiments and analysed the data. Yixin Zhou and Xin Zhong participated in the drafting of the article. All the authors agree to be accountable for all the aspects of this work and ensure its integrity and accuracy.

## CONFLICT OF INTEREST STATEMENT

The authors declare that they have no known competing financial interests or personal relationships that could have appeared to influence the work reported in this paper.

## ETHICS STATEMENT

All experimental procedures received approval from the Laboratory Animal Ethics Committee and the Laboratory Animal Centre of the First Affiliated Hospital of Wenzhou Medical University (no. WYYY‐IACUC‐AEC‐2021‐0262) and the Ethics Committee of the First Affiliated Hospital of Wenzhou Medical University (approval document no. KY2024‐R246).

## Supporting information



Supporting Information

## Data Availability

All the data needed to evaluate the conclusions in this study are presented in this manuscript or the Supporting Information. The materials described in this study are either commercially available or available upon reasonable request from the corresponding authors.
