# Supplementary material for: P2X7R deficiency alleviates cardiac senescence by enhancing mitophagy via the HuR/TRIM26/NR4A1 axis
Source: Clin Transl Med. 2026 Feb 26;16(3):e70621. doi: 10.1002/ctm2.70621 (PMC12940804; doi:10.1002/ctm2.70621)
Supplement: Supplementary file 1 — Supporting Information [file CTM2-16-e70621-s001.docx]

***Supplemental Appendix***

**P2X7R deficiency alleviates cardiac senescence by enhancing mitophagy via the HuR/TRIM26/NR4A1 axis**

**Contents in Supplementary File**

1、Extended Materials and Methods

2、Supplementary Table S1-5

3、Supplementary Figure S1-8 and Legends

**Extended Materials and Methods**

**Materials and Methods**

**Echocardiography**

Depilatory cream was used to remove the hair from the left precordial chest region. The mice were anaesthetised with isoflurane. Vevo LAB 3.1.1 was used to automatically calculate all cardiac function parameters, including left ventricular ejection fraction (EF), left ventricular fractional shortening (FS), left ventricular anterior wall thickness in diastole (LVAWd), and left ventricular anterior wall thickness in systole (LVAWs).

**RNA-seq analysis**

Total RNA was isolated from cardiac tissue specimens of young and senescent wild-type mice in compliance with the manufacturer's guidelines, utilizing TRIzol reagent (Invitrogen, cat. no. 15596018). The integrity of the RNA was assessed by denaturing agarose gel electrophoresis. Subsequently, poly(A) RNA was fragmented and subjected to reverse transcription with SuperScript™ II Reverse Transcriptase (Invitrogen, cat. no. 1896649) to generate cDNA. The ligated products were then amplified by PCR, yielding a final cDNA library with an average insert size of 300 ± 50 bp. The library was ultimately subjected to 2 × 150 bp paired-end sequencing on an Illumina NovaSeq™ 6000 system (LC-Bio Technology Co., Ltd., Hangzhou, China) according to the vendor's instructions.

**Cells and cultures**

HL-1 cells were obtained from the Cell Bank of the Shanghai Academy of Chinese Sciences (catalogue no. GNM 6) and were cultured in Dulbecco’s modified Eagle’s medium (DMEM, Thermo Fisher, #C11995500BT) containing 10% foetal bovine serum (Gibco, USA), 4.5 g/L glucose and 1% penicillin/streptomycin (100 U/mL penicillin and 100 mg/mL streptomycin) in a humidified CO_2_ incubator (5% CO2). The cells were utilised for experiments when they were in the exponential growth phase. Cardiomyocytes were pretreated with A-438079 (Selleck, #S7705), a P2X7R inhibitor, or DIM8 (Selleck, #S6799), an NR4A1 inhibitor, for 2 h before stimulation with D-gal (10 g/L).

**Sequential Extraction of Cytoplasmic and Nuclear Proteins**

The sequential extraction of cytoplasmic and nuclear proteins was performed with a Nuclear and Cytoplasmic Protein Extraction Kit (Beyotime, P0027) per the manufacturer's protocol. In brief, cultured cells were harvested, washed with ice-cold PBS, and subjected to lysis in a mild, non-ionic detergent buffer. After centrifugation, the supernatant constituting the cytoplasmic extract was collected. The insoluble nuclear pellet was then resuspended and lysed in a high-salt buffer to solubilize nuclear proteins. Fractionation purity was confirmed by Western blotting using GAPDH as a cytoplasmic marker and Histone H3 as a nuclear marker.

**Isolation and culture of primary cardiomyocytes**

Primary cardiomyocytes were isolated from the heart tissue of Sprague‒Dawley rats in accordance with established protocols. The hearts of Sprague‒Dawley neonatal rats (3 days) were removed and washed with phosphate-buffered saline (PBS) 3 times. The tissue was then cut, immersed in 10 ml of dissociation medium (trypsin and collagenase), and agitated slowly (approximately 15–20 times) for 8 min at 37°C to facilitate digestion. The fibroblasts were then subjected to the differential adhesion method, which involved the use of specific adhesion agents to separate and collect the desired cell types. Nonadherent cells were maintained in Dulbecco's modified Eagle's medium containing 10% foetal bovine serum and bromodeoxyuridine. Following a 48-h culture period, the cardiomyocytes were synchronised and utilised.

**Western blotting**

After heart tissues or cells were lysed in buffer (Boster Biological Technology, #AR0103/AR0101) containing a protein phosphatase inhibitor (Solarbio, #P1260), the samples were mixed with 5X dual-colour protein loading buffer (Fude Biological Technology, #FD006) and boiled for 10 min. After electrophoretic separation of the proteins via SDS‒PAGE, the proteins were transferred to a mixed cellulose ester transfer membrane (NC, Merck, #HATF00010). The membrane was subsequently blocked with 5% skim milk (Saiguo, #1172GR500) and then incubated with a primary antibody at 4°C for 8 h. The membrane was then incubated with horseradish peroxidase-labelled secondary antibodies for 1 h. The protein bands were visualized using Meilunbio®fg supersensitive ECL luminescence reagent (Meilunbio, #MA0186), and protein quantification was conducted using ImageJ software. A list of antibodies used in the study is available in the Supplemental Material (see Table 2).

**Real-time quantitative PCR**

Total RNA was isolated from heart tissues and HL-1 cells and purified using TRIzol (Thermo Fisher, #340312). RNA was reverse transcribed into cDNA using a RevertAid First Strand cDNA Synthesis Kit. RT–qPCR was performed with TB Green R Ex Tap TM II (TaKaRa, #RR820A) in a TANON 5000 chemiluminescence imageing system. The list of primer pairs used in the study is available in the Supplemental Material (see Table 1).

**Coimmunoprecipitation (co-IP)**

For the endogenous co-IP assay, protein was isolated from myocardial tissues or HL-1 cells using lysis buffer (Boster Biological Technology, #AR0103/AR0101). Then, protein A/G-coupled agarose beads (Beyotime, P2055) were added to the protein lysate, and the samples were rotated at 4°C for 2 h; a small proportion of the lysate was retained as input. The resulting supernatants were then gently mixed with primary antibodies, followed by rotation at 4°C for 8 h. Then, 20 μL of protein A/G-coupled agarose beads was added, followed by incubation at 4°C under gentle rotation for 2 h.

For the exogenous co-IP assay, HL-1 cells that had been transiently transfected with FLAG-TRIM26 plasmids were lysed in lysis buffer (Boster Biological Technology, #AR0103/AR0101). The lysate was centrifuged at 12,000 × g for 10 min at 4°C. The remaining steps were the same as those described above.

**RNA immunoprecipitation (RIP)**

An RNA binding protein immunoprecipitation (RIP) kit (Scbio, #KT102-0101) was used following the manufacturer’s instructions. In brief, 50 μl of protein A/G-coupled agarose beads were added to 1.5 ml DNA-free RNA-free centrifuge tubes (specific antibody group and IgG group), and the relevant primary antibodies, along with normal IgG, were added, followed by incubation at 4°C for 8 h with rotation. The samples were centrifuged at 1,000 rpm for 5 min. The upper layer was discarded, and 300 microlitres of heart tissue homogenate was added. The mixture was then incubated at 4°C for 8 h. The supernatant was discarded, and the beads were washed 5 times with RIP buffer. Finally, the immunoprecipitated RNA was extracted, and quantitative PCR was performed.

**Histological analysis**

Myocardial tissue was fixed in 4% paraformaldehyde, dehydrated (PFA), cleared, embedded in paraffin and sectioned at a thickness of 6 μm for subsequent experiments. In accordance with the manufacturer’s instructions, the sections were stained with haematoxylin and eosin (H&E) (Solarbio, #G1120), Masson (Solarbio, #G1340) and Sirius Red (Solarbio, #S8060), dehydrated in a gradient of ethanol, cleared with xylene and mounted with neutral balsam (Solarbio, cat no. G8590) solution. Finally, images were captured with an upright microscope (Leica, 400 × magnification) and analysed using ImageJ.

For frozen sections, myocardial tissues were preserved using Tissue-Tek O.C.T. compound (Sakura Finetek, #4583). The tissue blocks were subsequently frozen at -80 degrees Celsius, allowed to solidify, and then stored in liquid nitrogen. The blocks were sectioned (6 mm thick) and stored at -20°C for subsequent experiments. Then, the sections were stained with WGA (Solarbio, #I3300) in accordance with the manufacturer’s instructions and mounted with DAPI (Yeasen, 36308ES11). Finally, images were captured with an upright microscope (Leica, 400 × magnification) and analysed using ImageJ.

**Immunohistochemical staining**

The preparation steps were the same as previously described. Paraffin-embedded sections were soaked in sodium citrate buffer and subjected to antigen retrieval by microwaving. The sections were subsequently incubated with 3% H2O2 to inhibit endogenous peroxidase activity and with 5% bovine serum albumin (BSA; Solarbio, G8590) to block nonspecific binding prior to the addition of primary antibodies in blocking buffer (1:200). The sections were incubated overnight at 4°C and then washed 3 times with PBS, followed by incubation with HRP-conjugated secondary antibodies for 1 h. Visualisation was achieved via the use of a DAB Staining Kit (ZSJQ-BIO, #ZLI-9018), followed by counterstaining with haematoxylin. The remaining steps were the same as those described above.

**Immunofluorescence staining**

Frozen sections or cells were washed 3 times with PBS, followed by fixation in 4% PFA for 15 min. The samples were washed again with PBS and then permeabilized using 0.5% Triton X-100 in PBS for 20 min. Thereafter, the samples were incubated with primary antibodies (1:200) in blocking buffer overnight at 4°C. The next day, the samples were washed 3 times with PBS and then incubated with fluorescent secondary antibodies for 1 hour. Finally, the sections or cells were stained with DAPI (Yeasen, 36308ES11)to visualize nuclei, and images were captured with an upright microscope (Leica, 400× magnification) and analysed using ImageJ.

**SA-β-Gal staining**

A Senescence-Associated β-Galactosidase (SA-β-Gal) Stain Kit (Solarbio, #G1580) was used following the manufacturer’s instructions. The preparation steps for the frozen sections or cells were the same as previously described. After preparation, the sections or cells were incubated overnight with 1 mg/ml X-gal staining solution at 37°C. Images were captured using a microscope and analysed using ImageJ.

**Mitochondrial function assessment**

A mitochondrial membrane potential assay kit with JC-1 (Beyotime, #C2006) was used following the manufacturer’s instructions.

Mitochondrial morphology in the myocardia was observed by transmission electron microscopy (TEM). Myocardia were preserved in 2.5% glutaraldehyde for 18–20 h and then incubated for an additional hour in 1% osmium tetroxide. Heart tissues were then dehydrated and embedded in epoxy resin. Ultrathin sections (60 nm) were stained with 2% uranyl acetate and lead citrate. Ultrastructural visual fields were analysed using an electron microscope (HITACHI, H-7500).

**ROS and LPO measurements**

To assess ROS and oxidative stress levels, DHE (Beyotime, S0064S) and MitoSOX Red (Yeasen, #40778ES50) staining were used. For DHE fluorescence, HL-1 cells were incubated with DHE (1:100; diluted in PBS) for 30 min at 37°C. For MitoSOX Red, HL-1 cells were exposed to MitoSOX for 10 min at 37°C. Finally, DAPI was added dropwise to the cells, and images were captured by microscopy and analysed using ImageJ.

A Lipid Peroxidation MDA Assay Kit (Beyotime, #S0131) was used to measure the malondialdehyde (MDA) content, and a Total Superoxide Dismutase Assay Kit with WST-8 (Beyotime, #S0101S) was used to measure total superoxide dismutase (SOD) activity.

**mt-Keima Assay for Mitophagy Detection**

The mitophagic flux was monitored using the mitochondrial-targeted, pH-sensitive fluorescent probe mt-Keima. Cells were infected with a pre-packaged mt-Keima adenovirus (Genechem). Following a 24–48 hour incubation to allow for sufficient expression, the cells were treated according to the experimental design. Live-cell imaging was then performed using a confocal microscope equipped with 405 nm and 561 nm laser lines. The fluorescence emission at 620 nm was collected following sequential excitation at 440 nm and 561 nm. The ratio of the signal from excitation at 561 nm to that at 440 nm (561/440 nm ratio) was calculated on a pixel-by-pixel or per-cell basis using image analysis software (ImageJ). An increase in this ratio indicates the translocation of mt-Keima-labeled mitochondria into acidic lysosomes, confirming the occurrence of mitophagy.

**RNA stability assay**

HL-1 cells were incubated with actinomycin D (4 μM, Selleck, #S8964) and harvested at 0, 2, 4, 6, 8 and 10 h after being subjected to the indicated treatments. The remaining steps were the same as those for the qPCR analysis described above.

**Cell transfection**

Once the cells reached a confluence between 50% and 70%, a plasmid or specific small interfering RNA was transfected into the cells using Lipofectamine™ 3000 (Thermo Fisher Scientific, #L3000-015). After 24 h, the transfected cells were analyzed. Silencing of the TRIM26 gene in HL-1 cells was performed using a siRNA with the target sequence GAAGUUCUGGAUUGGGAAA. The following plasmids were obtained from Genechem: Flag-TRIM26 (mouse), Myc-NR4A1-WT (mouse), His-P2X7R (mouse).

**Supplementary Table S1**: Sequences of primers for real-time qPCR assay used in the study.

| Gene | Species | Sequence |
| --- | --- | --- |
| *Trim71* | Mouse | GCAGAAGTGAGCGACCAGCAG  CCGACTTGACCACGACCTTGAAG |
| *Trim41* | Mouse | GGAGGAGGAGGAGGAGGAGGAG  GTGGCGGCAGTTGGGTGATC |
| *Trim55* | Mouse | GAAGCCTGTGGTCATTCTCCCTTG  ACGGTGGTGCCTCCTCTTGTG |
| *Trim16* | Mouse | GGTCTATGCTGCTTTCTGGCTGTC  CTGGCTTCTCGGGCTCCTCTC |
| *Trim30a* | Mouse | ATGCCGAGTTCCTTACCCATTTGG  ATTCACCTTCTGCTCCTCCTCTGG |
| *Trim34a* | Mouse | AAGGAGCAGGAGAAGGTGGAGAC  TCTCAGTCTGGACCTGGCACTTC |
| *Trim65* | Mouse | GGACAACTCCTGGGCGTAGATTTG  AGGGAAGAGAGGCTGGCTGAAC |
| *Trim2* | Mouse | AAGCCTCTTTCGTGCCCAAACC  GTGCTGCTCCACCACATCCTTC |
| *Trim5* | Mouse | ACCATCGCCAGGGAACAAAGAAAG  GTTGAGCCTCTGTGACCTCTTGC |
| *Trim26* | Mouse | TACAGCAGCTACACGGCAGA  TCTTCAGGTAGGCGTTGTCG |
| *β-actin* | Mouse | CCAGATCCTGTCCAAACTAAGG  CTCTTTAGCATAGTAGTCCGCT |
| *P2x7r* | Mouse | GCTGGTGGTGGTTGTCATTG  AGGCGGATGATGAAGGTGAC |
| *Hur* | Mouse | CCTGGGAAACACTGTGGCTG  GGCGGTATTGGAACTTGTCG |
| *Il-1β* | Mouse | TCGCAGCAGCACATCAACAAGAG  AGGTCCACGGGAAAGACACAGG |
| *Il-6* | Mouse | TTCTTGGGACTGATGCTGGTGAC  GTGGTATCCTCTGTGAAGTCTCCTC |
| *Nr4a1* | Mouse | CCTTCAAAACCCAAGCAGCC  AAATTGTTGCACGTCACCGG |

**Supplementary Table S2**: antibody used in the study.

| Gene | Brand name | Product number |
| --- | --- | --- |
| GAPDH | HUABIO | ET1601-4 |
| P2X7R | HUABIO | ER1901-99 |
| P53 | HUABIO | ET1602-38 |
| P21 | HUABIO | HA500005 |
| P16 | HUABIO | ET1602-9 |
| P62 | HUABIO | HA721171 |
| PINK1 | HUABIO | HA723021 |
| Parkin | HUABIO | ET1702-60 |
| LC3B | HUABIO | ET1701-65 |
| TRIM26 | Santa | sc-393832 |
| HuR | HUABIO | ET1705-81 |
| Beta galactosidase | Proteintech | 15518-1-AP |

**Supplementary Table S3**: Baseline Clinical Characteristics.

| Variables | Total (n = 120) | Young life stage  (under 44, n = 40) | Middle life stage ( 45-59, n = 40) | Old life stage  (over 60, n = 40) | Statistic | *P* |  |
| --- | --- | --- | --- | --- | --- | --- | --- |
|  |  |  |  |  |  |  |  |
| BMI(kg/m^2^) | 22.966 ± 2.054 | 22.988 ± 3.054 | 23.046 ± 0.947 | 22.865 ± 1.622 | F=0.080 | 0.924 |  |
| Systolic blood pressure(mmHg) | 127.208 ± 14.243 | 121.700 ± 15.108 | 128.550 ± 13.105 | 131.375 ± 12.969 | F=5.227 | 0.007 |  |
| Diastolic blood pressure(mmHg) | 81.833 ± 10.799 | 82.200 ± 11.241 | 84.950 ± 10.066 | 78.350 ± 10.275 | F=3.957 | 0.022 |  |
| P2X7R(ng/ml) | 2.729 ± 2.094 | 0.680 ± 0.728 | 2.321 ± 0.715 | 5.184 ± 1.297 | F=228.909 | <.001 |  |
| Sex, n(%) |  |  |  |  | χ²=2.188 | 0.335 |  |
| Male | 83 (69.167) | 25 (62.500) | 31 (77.500) | 27 (67.500) |  |  |  |
| Female | 37 (30.833) | 15 (37.500) | 9 (22.500) | 13 (32.500) |  |  |  |
| Cigarette smoking, n(%) |  |  |  |  | - | 0.027 |  |
| current | 26 (21.667) | 8 (20.000) | 13 (32.500) | 5 (12.500) |  |  |  |
| never | 80 (66.667) | 30 (75.000) | 19 (47.500) | 31 (77.500) |  |  |  |
| past | 14 (11.667) | 2 (5.000) | 8 (20.000) | 4 (10.000) |  |  |  |
| Hypertension, n(%) |  |  |  |  | χ²=12.890 | 0.002 |  |
| no | 69 (57.500) | 32 (80.000) | 20 (50.000) | 17 (42.500) |  |  |  |
| yes | 51 (42.500) | 8 (20.000) | 20 (50.000) | 23 (57.500) |  |  |  |
| Hypertension drugs, n(%) |  |  |  |  | χ²=12.890 | 0.002 |  |
| no | 69 (57.500) | 32 (80.000) | 20 (50.000) | 17 (42.500) |  |  |  |
| yes | 51 (42.500) | 8 (20.000) | 20 (50.000) | 23 (57.500) |  |  |  |
| Diabetes, n(%) |  |  |  |  | - | 1.000 |  |
| no | 116 (96.667) | 39 (97.500) | 39 (97.500) | 38 (95.000) |  |  |  |
| yes | 4 (3.333) | 1 (2.500) | 1 (2.500) | 2 (5.000) |  |  |  |
| Previous heart failure, n(%) |  |  |  |  | - | 0.328 |  |
| no | 118 (98.333) | 40 (100.000) | 40 (100.000) | 38 (95.000) |  |  |  |
| yes | 2 (1.667) | 0 (0.00) | 0 (0.00) | 2 (5.000) |  |  |  |
| CRP , n(%) |  |  |  |  | χ²=14.118 | <.001 |  |
| CRP<10 mg/L | 102 (85.000) | 40 (100.000) | 34 (85.000) | 28 (70.000) |  |  |  |
| CRP≥10mg/L | 18 (15.000) | 0 (0.00) | 6 (15.000) | 12 (30.000) |  |  |  |
| Data are mean ± SD or number(%) of patients  F: ANOVA, χ²: Chi-square test, -: Fisher exact | | | | | | |  |
| CRP:C-reactive protein | | | | | | | ； |

**Supplementary Table S4**:Association between P2X7a levels and age: univariable and multivariable linear regression models

| Variables | Univariate | | | | |  | Multivariate | | | | |
| --- | --- | --- | --- | --- | --- | --- | --- | --- | --- | --- | --- |
|  | β | S.E | t | *P* | β (95%CI) |  | β | S.E | t | *P* | β (95%CI) |
| Sex |  |  |  |  |  |  |  |  |  |  |  |
| Male |  |  |  |  | 0.000 (Reference) |  |  |  |  |  |  |
| Female | -0.210 | 0.415 | -0.505 | 0.615 | -0.210 (-1.024 ~ 0.604) |  |  |  |  |  |  |
| Cigarette smoking |  |  |  |  |  |  |  |  |  |  |  |
| current |  |  |  |  | 0.000 (Reference) |  |  |  |  |  |  |
| never | 0.286 | 0.476 | 0.602 | 0.548 | 0.286 (-0.647 ~ 1.219) |  |  |  |  |  |  |
| past | 0.139 | 0.699 | 0.199 | 0.843 | 0.139 (-1.231 ~ 1.509) |  |  |  |  |  |  |
| Hypertension |  |  |  |  |  |  |  |  |  |  |  |
| no |  |  |  |  | 0.000 (Reference) |  |  |  |  |  |  |
| yes | 0.746 | 0.382 | 1.950 | 0.054 | 0.746 (-0.004 ~ 1.495) |  |  |  |  |  |  |
| Hypertension drugs |  |  |  |  |  |  |  |  |  |  |  |
| no |  |  |  |  | 0.000 (Reference) |  |  |  |  |  |  |
| yes | 0.746 | 0.382 | 1.950 | 0.054 | 0.746 (-0.004 ~ 1.495) |  |  |  |  |  |  |
| Diabetes |  |  |  |  |  |  |  |  |  |  |  |
| no |  |  |  |  | 0.000 (Reference) |  |  |  |  |  |  |
| yes | 0.025 | 1.070 | 0.023 | 0.982 | 0.025 (-2.072 ~ 2.121) |  |  |  |  |  |  |
| Previous heart failure |  |  |  |  |  |  |  |  |  |  |  |
| no |  |  |  |  | 0.000 (Reference) |  |  |  |  |  |  |
| yes | 2.630 | 1.480 | 1.777 | 0.078 | 2.630 (-0.271 ~ 5.531) |  |  |  |  |  |  |
| CRP |  |  |  |  |  |  |  |  |  |  |  |
| CRP<10 mg/L |  |  |  |  | 0.000 (Reference) |  |  |  |  |  | 0.000 (Reference) |
| CRP≥10mg/L | 1.635 | 0.516 | 3.168 | 0.002 | 1.635 (0.624 ~ 2.647) |  | 0.112 | 0.333 | 0.336 | 0.738 | 0.112 (-0.541 ~ 0.764) |
| Age | 0.099 | 0.007 | 15.113 | <.001 | 0.099 (0.086 ~ 0.111) |  | 0.098 | 0.007 | 14.137 | <.001 | 0.098 (0.084 ~ 0.111) |
| BMI | -0.034 | 0.094 | -0.367 | 0.714 | -0.034 (-0.218 ~ 0.149) |  |  |  |  |  |  |
| Systolic blood pressure | 0.025 | 0.013 | 1.869 | 0.064 | 0.025 (-0.001 ~ 0.051) |  |  |  |  |  |  |
| Diastolic blood pressure | -0.032 | 0.018 | -1.834 | 0.069 | -0.032 (-0.067 ~ 0.002) |  |  |  |  |  |  |
| CI: Confidence Interval  CRP:C-reactive protein | | | | | | | | | | | |

**Supplementary Table S5**:Subgroup analysis of the association between the age and P2X7R serum levels

| Variables | n (%) | β (95%CI) | *P* | P for interaction |
| --- | --- | --- | --- | --- |
|  |  |  |  |  |
| All patients | 120 (100.00) | 0.10 (0.09 ~ 0.11) | <.001 |  |
| Sex |  |  |  | 0.169 |
| Male | 83 (69.17) | 0.11 (0.09 ~ 0.12) | <.001 |  |
| Female | 37 (30.83) | 0.09 (0.07 ~ 0.11) | <.001 |  |
| Hypertension |  |  |  | 0.168 |
| No | 69 (57.50) | 0.10 (0.09 ~ 0.12) | <.001 |  |
| Yes | 51 (42.50) | 0.12 (0.10 ~ 0.15) | <.001 |  |
| Diabetes |  |  |  | 0.743 |
| No | 116 (96.67) | 0.10 (0.09 ~ 0.11) | <.001 |  |
| Yes | 4 (3.33) | 0.09 (0.06 ~ 0.12) | 0.031 |  |
| CRP |  |  |  | 0.653 |
| CRP<10 mg/L | 102 (85.00) | 0.10 (0.08 ~ 0.11) | <.001 |  |
| CRP≥10mg/L | 18 (15.00) | 0.09 (0.04 ~ 0.13) | 0.002 |  |
| CI: Confidence Interval  CRP:C-reactive protein | | | | |

***Supplementary Figures:***


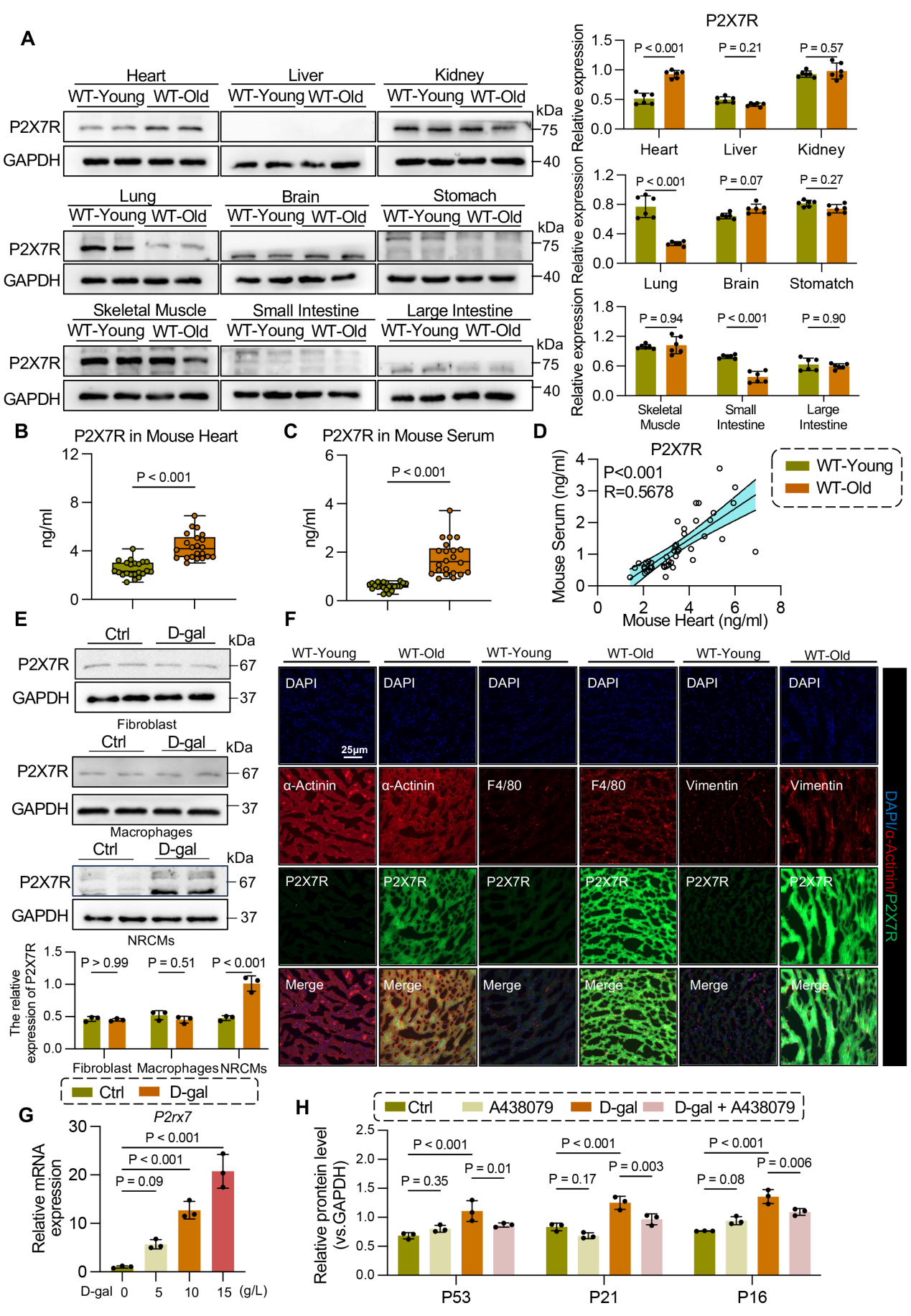


**Figure S1: A** (n=6), P2X7R expression in heart, liver, kidney, lung, brain, stomach, skeletal muscle, small intestine, large intestine of WT-Young and WT-Old mice. **B**(n=22), P2X7R expression in myocardial tissue of WT-Young and WT-Old group.**C**(n=22), P2X7R expression in mouse serum of WT-Young and WT-Old group. **D**(n=44), Correlation between P2X7R expression in plasma and in myocardial tissue. **E** (n=3), P2X7R expression in fibroblast,macrophages and primary cardiomyocytes induced by D-gal.**F**, Double immunofluorescence staining for P2X7R (green), the fibrosis marker vimentin (red) or the myocyte marker α-actinin (red) or

pan-macrophage marker F4/80(red) in the myocardium of WT-Young and WT-Old mice; Merged images (orange) showing colocalization; scale bar, 25 μm. **G** (n=3), mRNA levels of P2X7R in HL-1 cells treated by 0，5，10，15g/L D-gal. **H** (n=3), Densitometric quantification of immunoblots in **Figure 1K**.


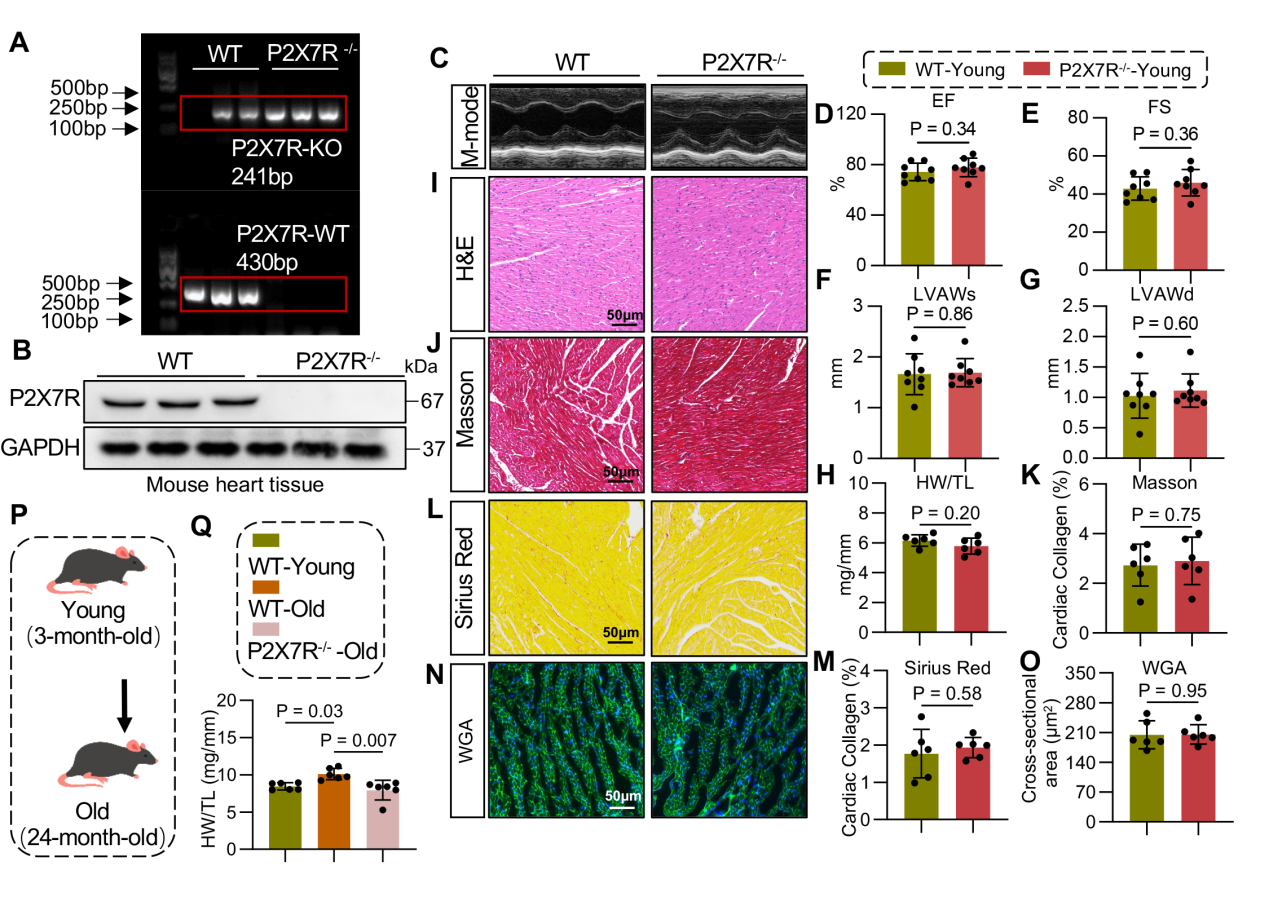


**Figure S2: A**, Images of gene identification from male P2X7R knockout mice (P2X7R^-/-^) and their control wild-type (WT) littermates.**B**(n=6), Verification of ablation of P2X7R in heart. **C**, Representative M-mode echocardiographic images of the left ventricle from WT-Young or P2X7R^-/-^-Young mice. **D and E** (n=8), LV ejection fraction (EF) and fractional shortening (FS) were assessed by echocardiography. **F and G** (n=8), Left ventricular anterior wall tickness(LVAWs and LVAWd) were assessed by echocardiography. **H** (n=6), the ratio of heart weight/tibia length (HW/TL) of WT-Young or P2X7R^-/-^-Young mice. **I**, Representative images of H&E-stained; scale bar, 50 μm. **J and K** (n=6), Representative images of Masson staining **(J)**; scale bar, 50 μm and quantification of the interstitial fibrotic area (**K)**. **L and M** (n=6), Representative images of Sirius Red **(L)**; scale bar, 50 μm and quantification of the interstitial fibrotic area **(M)**. **N and O** (n=6), Representative images of WGA-stained sections **(N)**; scale bar, 50 μm and quantification of cardiomyocyte cross-sectional area **(O)**. **P**, C57BL/6 mice were fed a normal diet for 3 months or 24 months to bulid aging animal model. **Q** (n=6), the ratio of weight/tibia length (HW/TL) of WT-young, WT-old or P2X7R^-/-^-old mice.


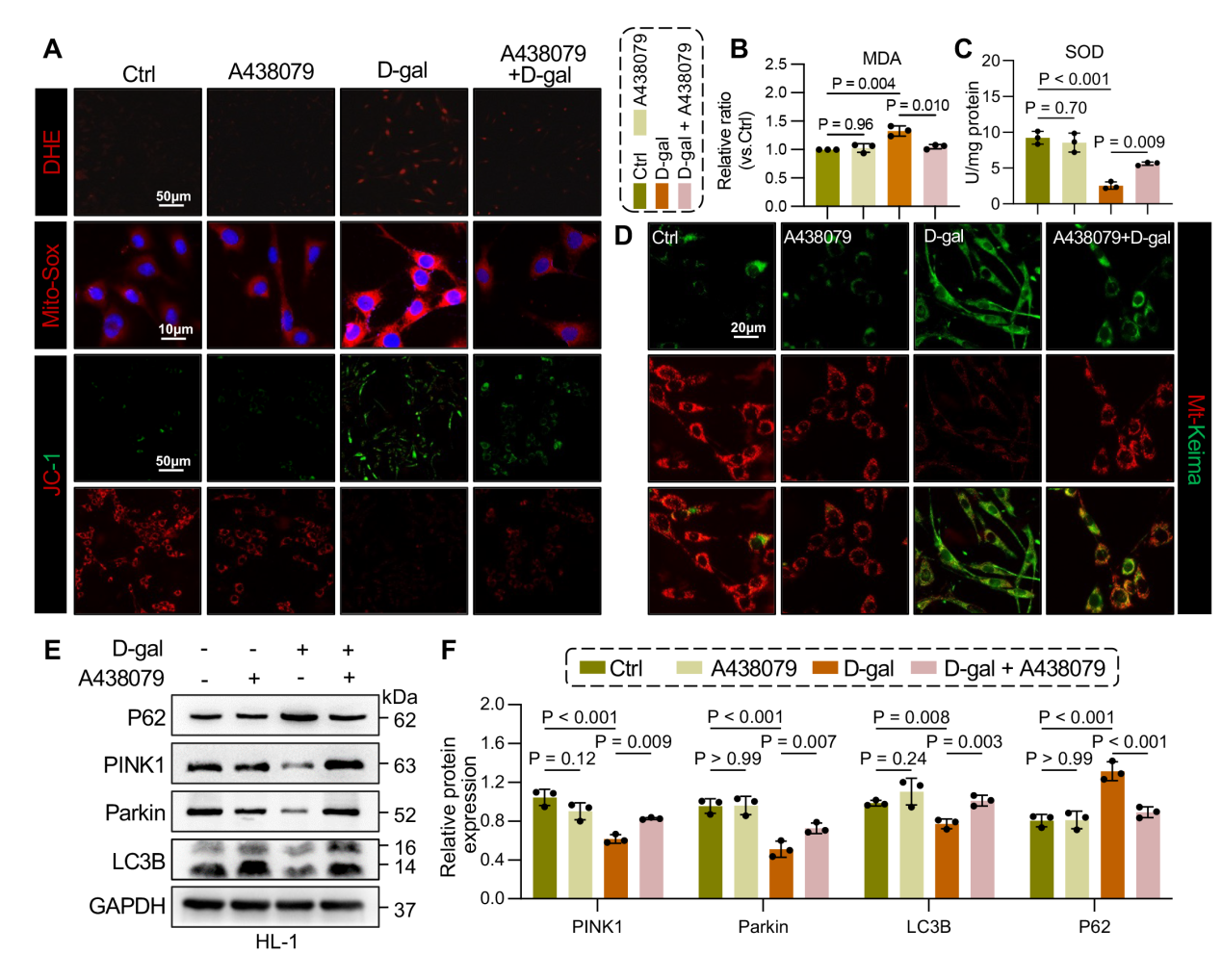


**Figure S3: A**, Mitochondrial membrane potential of HL-1 cells was measured by DHE staining (scale bar, 50 μm), Mito-Sox staining (scale bar, 10 μm) and JC-1 staining (scale bar, 50 μm). **B** and **C** (n=3), Representative MDA content **(B)** and SOD activity **(C)** in Ctrl, A438079, D-gal and A438079+D-gal group. **D,** Representative confocal images of Ctrl, A438079, D-gal and A438079+D-gal group expressing mt-Keima; scale bar, 20 μm. **E** (n=3), Representative Western blot analysis of P62, PINK1, Parkin and LC3B levels in Ctrl, A438079, D-gal and A438079+D-gal group. GAPDH was used as a loading control. **F** (n=3), Densitometric quantification of immunoblots in **E**.

**
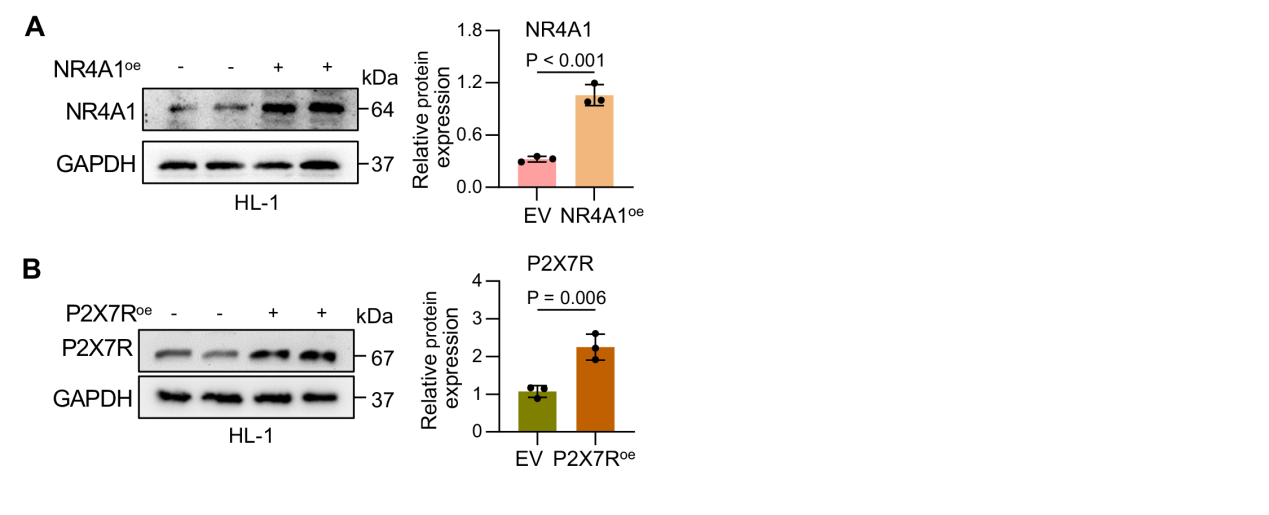
**

**Figure S4: A** (n=3), NR4A1 expression in HL-1 cells transfected with Myc-NR4A1, GAPDH was used as a loading control and density analysis. **B** (n=3), P2X7R expression in HL-1 cells transfected with His-P2X7R, GAPDH was used as a loading control and density analysis.

**
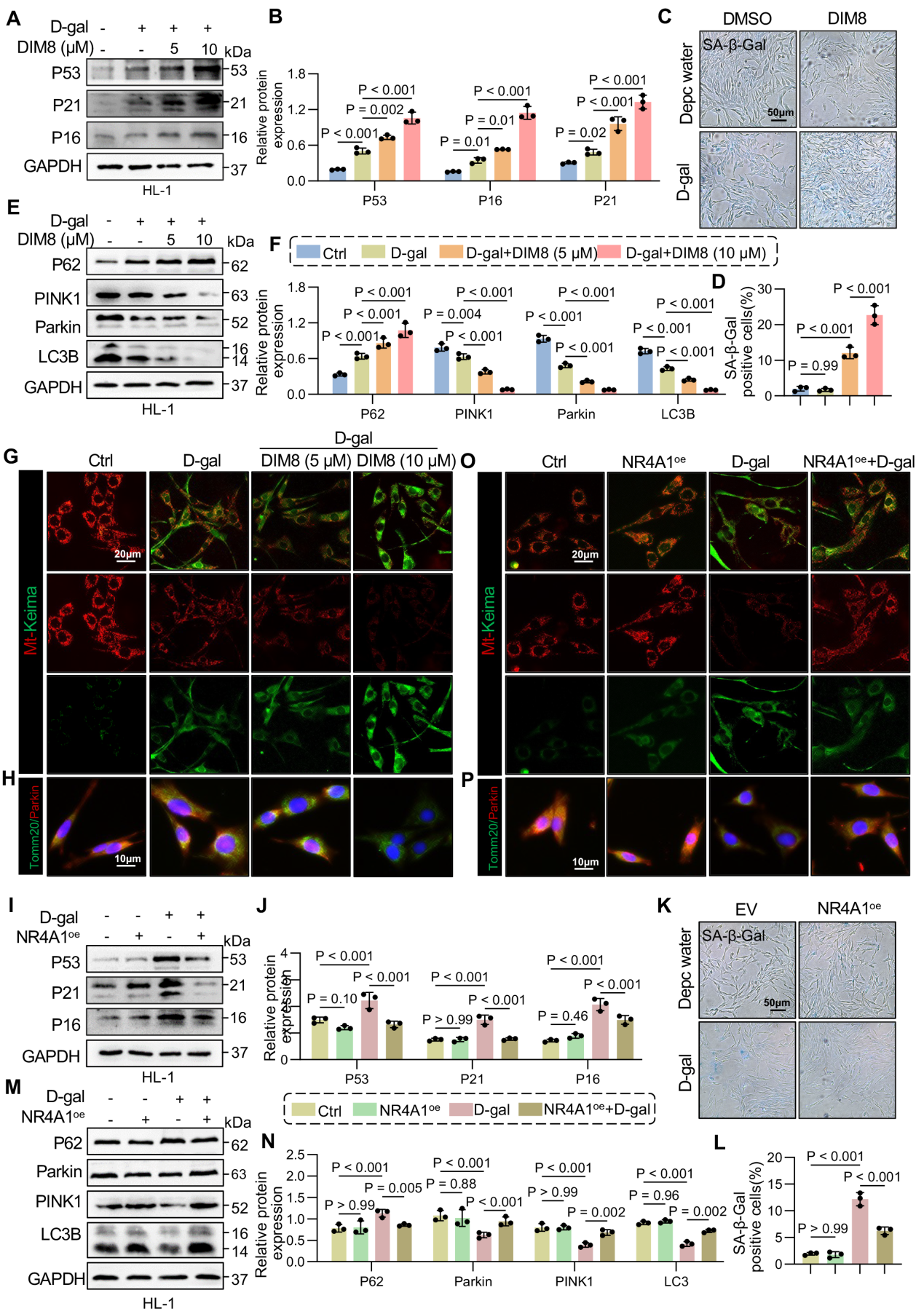
**

**Figure S5**: **A and B** (n=3), Representative Western blot analysis of P53,P21 and P16 levels in HL-1 cells treated with D-gal or DIM8 **(A)**, GAPDH was used as a loading control and density analysis **(B)**. **C and D** (n=3), Representative images of SA-β-Gal staining in HL-1 cells treated with D-gal or DIM8 **(C)**; scale bar, 50 μm and quantification of percentages of SA-β-Gal+ area **(D)**. **E and F** (n=3), Representative Western blot analysis of P62, PINK1, Parkin and LC3B levels in HL-1 cells treated with D-gal or DIM8, GAPDH was used as a loading control **(E)** and density analysis **(F)**. **G**, Representative confocal images of HL-1 cells treated with D-gal or DIM8 expressing mt-Keima; scale bar, 20 μm. **H**, Double immunofluorescence staining for Parkin (red), TOMM20 (green) in the HL-1 cells treated with D-gal or DIM8. Merged images (orange) showing colocalization; scale bar, 20 μm. **I and J** (n=3), Representative Western blot analysis of P53, P21 and P16 levels in HL-1 cells treated with D-gal or NR4A1^oe^, GAPDH was used as a loading control **(I)** and density analysis **(J)**. **K and L**(n=3), Representative images of SA-β-Gal staining in HL-1 cells treated with D-gal or NR4A^oe^ **(K)**; scale bar, 50 μm and quantification of percentages of SA-β-Gal+ area **(L)**. **M and N** (n=3), Representative Western blot analysis of P62, PINK1, Parkin and LC3B levels in HL-1 cells treated with D-gal or NR4A1^oe^, GAPDH was used as a loading control **(M)**and density analysis **(N)**. **O**, Representative confocal images of HL-1 cells treated with D-gal or NR4A1^oe^ expressing mt-Keima; scale bar, 20 μm. **P,**Double immunofluorescence staining for Parkin (red), TOMM20 (green) in the HL-1 cells treated with D-gal or NR4A1^oe^ . Merged images (orange) showing colocalization; scale bar, 20 μm. Adjusted P values were provided in case of multiple groups. DIM8 = an inhibitor of NR4A1.

**
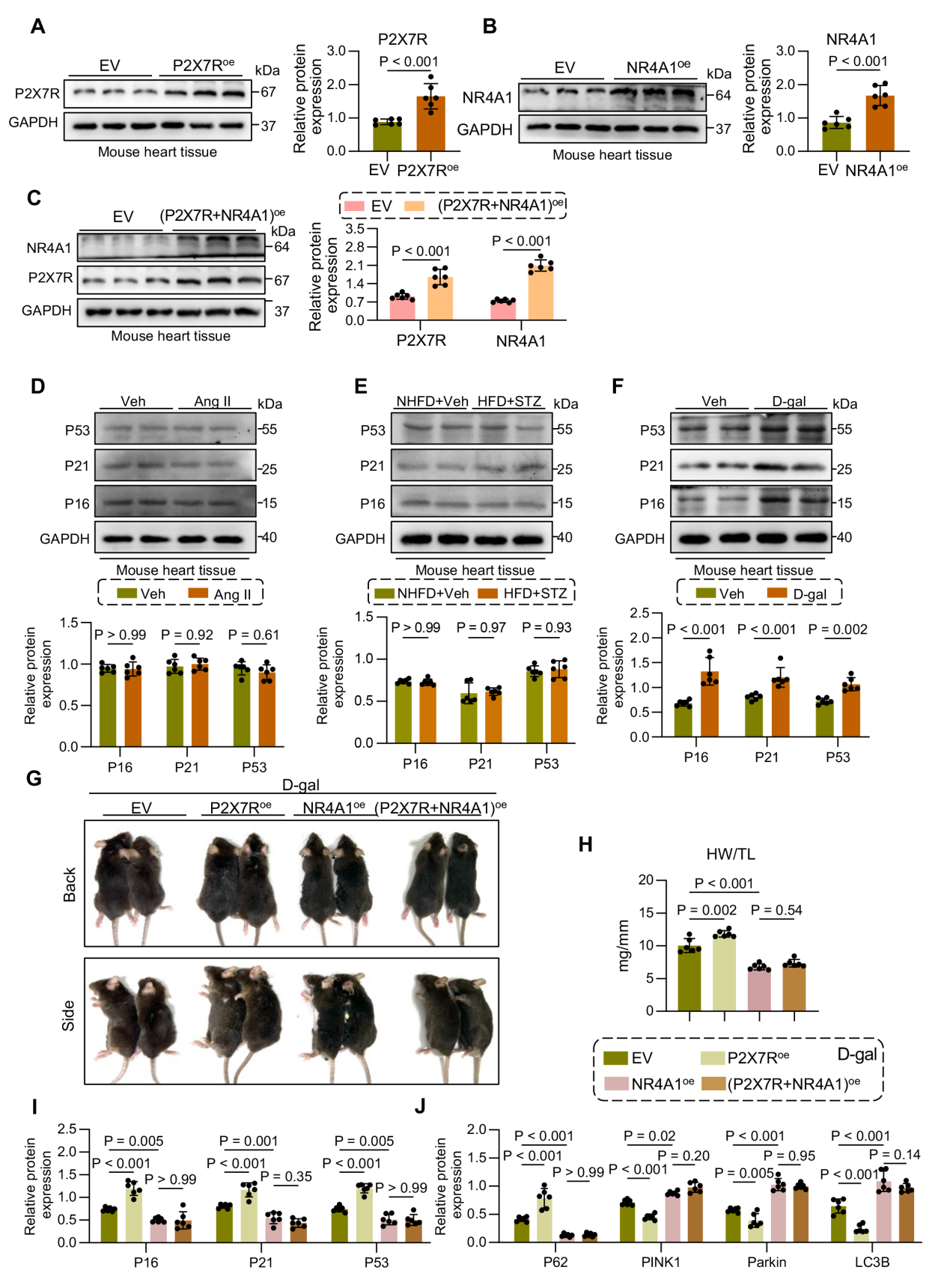
**

**Figure S6: A** (n=6), Expression of P2X7R in heart tissue of mice that received rAAV9-null or rAAV9-cTnT-P2X7R. **B** (n=6), Expression of NR4A1 in heart tissue of mice that received rAAV9-null or rAAV9-cTnT-NR4A1.**C** (n=6), Expression of NR4A1 and P2X7R in heart tissue of mice that received rAAV9-null or rAAV9-cTnT-NR4A1 and rAAV9-cTnT-P2X7R. **D-F** (n=6), Representative western blot analysis of P53, P21 and P16 levels in mouse heart tissue following induction by Ang II **(D)**, HFD+STZ **(E)**, and D-gal **(F)**, GAPDH was used as a loading control and density analysis. **G**, Representative image of D-gal+EV, D-gal+P2X7R^oe^, D-gal+NR4A1^oe^ or D-gal+P2X7R^oe^+ NR4A1^oe^ mice. **H** (n=6), the ratio of heart weight/tibia length (HW/TL) in each group of mice. **I** (n=6), Densitometric quantification of immunoblots in **Figure 5P**. **J**(n=6), Densitometric quantification of immunoblots in **Figure 5Q**.


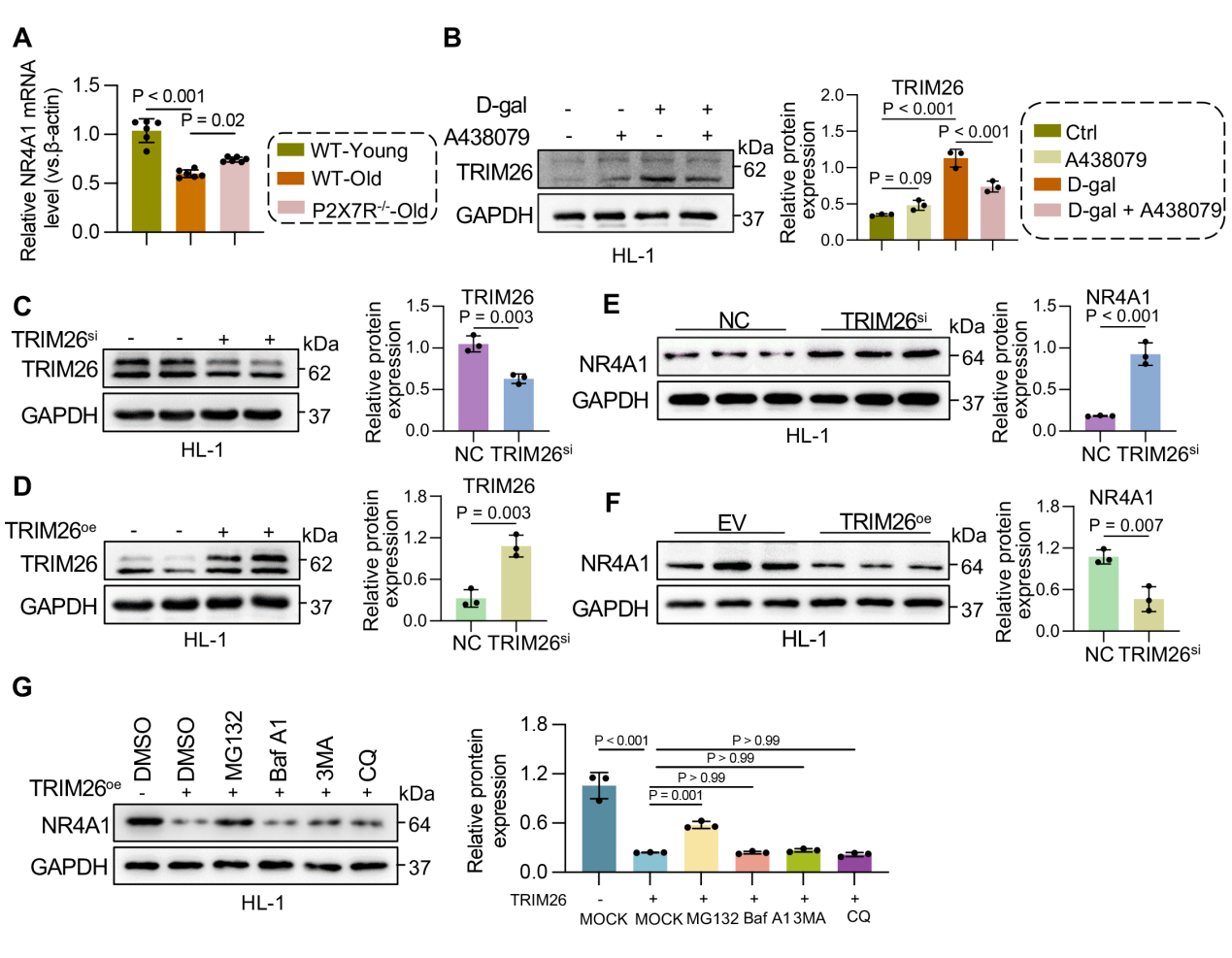


**Figure S7:** **A**(n=3), NR4A1 mRNA expression in myocardial tissue of WT-Young, WT-Old or P2X7R^-/-^-Old mice. **B** (n=3), TRIM26 expression in D-gal indeced HL-1 cells, GAPDH was used as a loading control and density analysis. **C and D**(n=3), TRIM26 expression in HL-1 transfected with si-TRIM26 **(C)**and TRIM26 plasmid**(D)**, GAPDH was used as a loading control and density analysis. **E** (n=3), NR4A1 expression in HL-1 cells transfected with si-TRIM26, GAPDH was used as a loading control and density analysis. **F** (n=3), NR4A1 expression in HL-1 cells transfected with Flag-TRIM26, GAPDH was used as a loading control and density analysis.**G** (n=3), Protein level of NR4A1 in HL-1 cardiomyocytes subjected to proteasome inhibitor (MG132, 10 mM) or autophagy inhibitors (3-MA, 10 mM; CQ, 50 mM and Baf A1, 200 nM) and density analysis. Adjusted P values were provided in case of multiple groups. 3-MA = 3-methyladenine; Baf A1 = Bafilomycin A1; CHX = cycloheximide; CQ = Chloroquine.


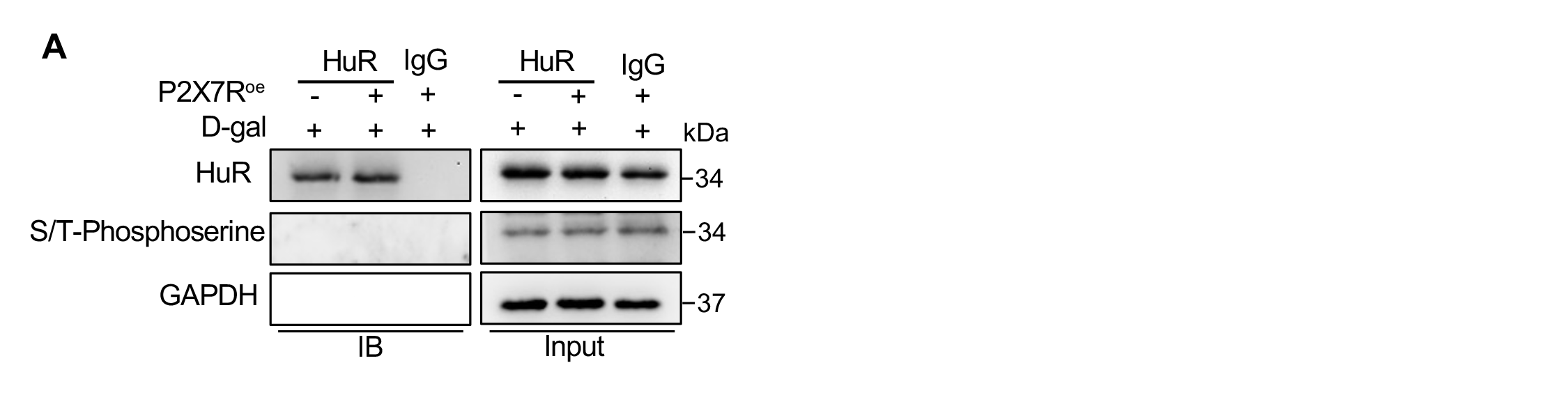


**Figure S8:A**, Co-IP of HuR and S/T-Phosphoserine in cardiomyocytes induced by D-gal and His-P2X7R. Endogenous HuR was immunoprecipitated by anti-HuR.
